# TENSO: Software Package for Numerically Exact Open Quantum Dynamics Based on Efficient Tree Tensor Network Decomposition of the Hierarchical Equations of Motion

**DOI:** 10.1021/acs.jctc.6c00525

**Published:** 2026-07-15

**Authors:** Juan C. Rodriguez-Betancourt, Michelle C. Anderson, Luchang Niu, Xinxian Chen, Ignacio Franco

**Affiliations:** † Department of Chemistry, 6927 University of Rochester, Rochester, New York 14627, United States; ‡ Department of Physics and Astronomy, 1438 University of Rochester, Rochester, New York 14627, United States; § The Institute of Optics, University of Rochester, Rochester, New York 14627, United States

## Abstract

TENSO is a versatile and powerful open-source software package for numerically exact simulations of the dynamics of quantum systems immersed in structured thermal environments. It is based on a tree tensor network decomposition of the hierarchical equations of motion (HEOM) that efficiently curtails its curse of dimensionality with bath complexity. As such, TENSO enables exact non-Markovian open-quantum-dynamics simulations even with complex environments typical of chemistry and quantum information science. TENSO allows for time-dependent drive in the system and for noncommuting fluctuations. More generally, TENSO efficiently propagates the dynamics for any method with a generator of the dynamics that can be expressed in a sum-of-products form, including the HEOM and multilayer multiconfigurational time-dependent Hartree methods. TENSO enables simulations using tensor trees and trains of arbitrary order and implements three propagation strategies for the coupled master equations: two fixed rank methods that require a constant memory footprint during the dynamics and one adaptive rank method with a variable memory footprint controlled by the target level of computational error. In contrast to the accompanying theory and algorithmic paper [ J. Chem. Phys. 2025, 163, 104109 40919968 ], the focus here is on the practical usage and applications of TENSO with underlying theoretical concepts introduced only as needed.

## Graphical abstract

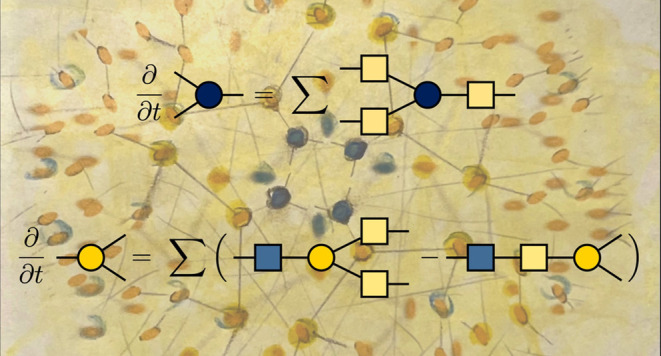

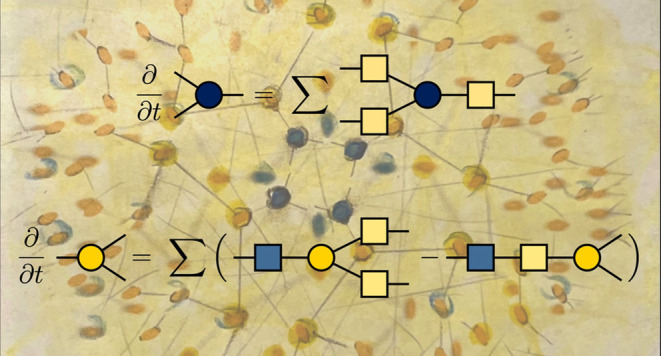

## 1 Introduction

Open quantum systems refer to systems interacting with their quantum environment. This interaction introduces quantum noise or decoherence and is responsible for ubiquitous processes in nature such as dephasing, energy relaxation, and the thermalization of quantum systems, making a detailed understanding of open quantum dynamics essential in chemistry, physics, and quantum information science.^1–7^ Further, elucidating decoherence mechanisms is necessary to engineer quantum environments for quantum and chemical control tasks.^8–13^ The aim of TENSO, Tensor Equations for Non-Markovian Structured Open Systems,^14,15^ is to predict the dynamics of quantum systems interacting with macroscopic thermal environments of arbitrary complexity using the numerically exact hierarchical equations of motion (HEOM).^16^ TENSO combines the bexcitonic generalization of the HEOM^17^—which unites HEOM variants and exposes their mathematical structure—with a tree tensor network (TTN) decomposition, which enables numerically efficient simulation.

In open quantum dynamics, it is customary to decompose the Hamiltonian, *H* = *H*_S_ + *H*_SB_ + *H*_B_, of the quantum universe into Hamiltonians of a system *H*_S_, a bath *H*_B_, and their interaction *H*_SB_.^1^ Since the bath is usually macroscopic, the strategy in open quantum dynamics is to develop quantum master equations (QMEs) that capture its influence on the system without propagating the bath degrees of freedom explicitly. A variety of approaches have been developed to simulate open quantum system dynamics.^1,6,7,18–22^ Of particular interest are numerically exact QMEs as they can be used to model a large class of problems of importance in chemistry and quantum information science with an accuracy that can be assured. This contrasts with common strategies based on the Born-Markov approximation that only apply to systems weakly coupled to fast environments with short memory time,^1^ conditions that are often too restrictive for chemical dynamics.

The HEOM is a powerful method for numerically exact open quantum dynamics for systems embedded in thermal Bosonic environments.^16,17,23–25^ In the case of linear system-bath coupling, the bath correlation function (BCF) contains all information about the bath needed to construct the quantum master equation. To make the QME numerically tractable, HEOM decomposes the BCF into a series of *K* complex decaying exponentials which allows the influence of the environment to be captured by a hierarchy of auxiliary density matrices (ADMs). The HEOM has been employed effectively for models of spectroscopy, and for charge and energy transport in photosynthetic complexes, molecular aggregates, Holstein models, and organic photovoltaic cells.^26–32^ The main challenge of HEOM is that the cost of the quantum dynamics grows exponentially with the number of features *K* in the bath, thus limiting the use of HEOM to simple model problems with unstructured environments.

Due to its importance, several computational packages implement HEOM simulations, including QuTiP, pyHEOM, HierarchicalEOM.jl, GPU-HEOM, and DM-HEOM.^23,27,33–35^ However, these implementations are often limited by the number of features *K* that can be included, and present-day capabilities are insufficient to capture chemically realistic environments, especially when low-temperature correction terms are required.

To mitigate this curse of dimensionality, recently we introduced a TTN decomposition of the HEOM (TTN-HEOM), which allows for efficient compression of the simulation space, thus enabling simulations of open quantum systems interacting with environments with large numbers of features.^14^ Our approach further allows for time-dependent system Hamiltonians and multiple baths, providing a useful and versatile method for investigating dissipative dynamics.

Tensor network decompositions represent the state of the art in the simulation of many-body systems.^36–43^ In particular, they are the basis for the powerful multilayer multiconfigurational time-dependent Hartree (ML-MCTDH)^37,44,45^ method, which is the gold standard in high-dimensional closed quantum dynamics simulations. Our TTN-HEOM developments are parallel to the ML-MCTDH as the three main principles (sum of product dynamical generator, TTN decomposition, and Dirac-Frenkel time-dependent variational principle (TDVP)^44,46–48^ to isolate the equations of motion) are identical. However, while ML-MCTDH is designed for unitary dynamics, the TTN-HEOM is designed for thermal dissipative dynamics.

TENSO employs tensor network approaches by arranging the large set of ADMs in the HEOM formalism into an extended density operator (EDO). This higher-order EDO is decomposed into core tensors using the TTN representation. To optimally evolve the TTN such that it correctly represents the EDO, TENSO applies the TDVP to develop the QMEs for the core tensors. The TENSO implementation of this QME provides efficient, stable, error-controlled evolution that is best suited for open quantum dynamics with strong coupling and highly structured bath spectral densities.

We illustrate the usage of TENSO and demonstrate its versatility and wide applicability to open quantum dynamics through three examples. First is the quintessential spin-boson problem, which is a key model in spectroscopy and quantum information.^19,49,50^ Using this model, we will illustrate the fundamental features of TENSO, how to perform simulations with structured spectral densities, how TENSO easily addresses noncommuting fluctuations, how to invoke time-dependent Hamiltonians, and how to manipulate the convergence. We will also briefly mention TENSO’s extension to MCTDH, emphasizing the adaptability of TENSO’s underlying structure to other master equations with propagators in sum-of-products form. We then address the Fenna-Matthews-Olson (FMO)^51,52^ complex, a paradigmatic multilevel model problem in the study of photosynthetic energy capture.^53–58^ We use it to demonstrate how to employ TENSO to address multilevel systems coupled to multiple structured baths. Our third example is the sudden death of entanglement between two qubits interacting with a thermal environment.^59^ We use it to demonstrate how TENSO can address problems in quantum information.

It is useful to frame TENSO within the increasing ecosystem of tensor-network-based simulation methods. The packages pyTTN^45^ and QuTree^60^ provide frameworks for tensor operations. TTNO^61^ yields an optimized sum-of-products decomposition of general operators. RENORMALIZER^62^ and MPSDynamics.jl^63^ provide tensor-based time-dependent density matrix renormalization group (TD-DMRG) and time-evolving density operator with orthogonal polynomials (TEDOPA) computations.^64^ The Heidelberg MCTDH package focuses on wave-function-based dynamics.^65^ In contrast, TENSO TENSOis focused on the HEOM calculations. The mpsqd^66^ also implements tensor-based HEOM but using tensor trains instead of tensor trees. Together, TENSO’s HEOM focus, accessible Python implementation, and ability to address general tree structures with both adaptive and fixed rank propagation strategies differentiate it from other options and make it a versatile tool in this ecosystem.

The structure of this paper is as follows. Section 2 reviews the bexcitonic HEOM and its TTN decomposition. Section 3 demonstrates the installation, usage, and capabilities of TENSO through representative numerical examples. Section 4 summarizes typical simulation parameters appropriate for most problems, and Section 5 discusses computational cost. Section 6 provides information on the architecture and design of the TENSO package, and Section 7 discusses modifications that can be made to the TENSO code to address specialized or more advanced applications.

## 2 Methods

We now provide an overview of the most important theory underlying TENSO. References 14 and 17 provide detailed derivations and a full discussion of the propagation strategies employed. Throughout, we use atomic units where ℏ = 1.

### 2.1 Bath Correlation Function and Its Decomposition into Features

The HEOM focuses on the open quantum dynamics of systems immersed in an environment that can be described as a collection of harmonic oscillators with Hamiltonian

1 $$H_{B}=\sum_{j}\left(\frac{p_{j}^{2}}{2m_{j}}+\frac{m_{j}\omega_{j}^{2}x_{j}^{2}}{2}\right)$$

Here, *x*_*j*_ and *p*_*j*_ are the position and momentum of the *j*-th oscillator with frequency ω_*j*_ and effective mass *m*_*j*_. The system-bath coupling Hamiltonian is of the form *H*_SB_ = *Q*_S_ ⊗ *X*_B_, where *Q*_S_ is a system operator and *X*_B_ = ∑_*j*_ *c*_*j*_ *x*_*j*_ is a collective bath coordinate. Here, *c*_*j*_ quantifies the coupling strength between the system operator and the *j*th bath mode. Harmonic models of the environment are broadly used as any environment can be represented by this model up to second order in perturbation theory.^67,68^ Further, this is commonly applicable in the thermodynamic limit as the system-bath interaction is diluted over a macroscopic number of modes.^69,70^ For harmonic environments, the dynamical properties of the bath are completely captured by the BCF, *C*(*t*) = ⟨*X̃*_B_(*t*)*X̃*_B_(0)⟩, where *X̃*_B_(*t*) is the collective bath coordinate in the interaction picture of *H*_S_ + *H*_B_ and the expectation value is over the bath thermal state.^19^ Formally, the BCF can be expressed as

2 $$C\left(t\right)=\int_{-\infty }^{+\infty }J\left(\omega \right)f_{\mathrm{BE}}\left(\beta \omega \right)e^{-i\omega t}d\omega$$

where

3 $$J\left(\omega \right)=\sum_{j}\frac{|c_{j}{|}^{2}}{\left(2m_{j}\omega_{j}\right)}\left[\delta \left(\omega -\omega_{j}\right)-\delta \left(\omega +\omega_{j}\right)\right]$$

is an odd extension of the bath spectral density, a quantity that summarizes the frequencies {ω_*j*_} of the oscillators in the bath and specifies their coupling strengths to the system. The function *f*_BE_(*βω*) = (1 – *e* ^–*βω*^)^−1^ is related to the Bose–Einstein distribution, and β = 1/*k*_B_ *T* is the inverse temperature. Without loss of generality, the residue theorem is used to decompose the BCF into a sum of complex exponential functions referred to as features, typically via Padé^71^ or Matsubara^72^ expansions. This allows us to write the BCF as

4 $$C\left(t\right)=-2\pi i\sum_{i}{\mathrm{Res}}_{z=\zeta_{i}}\left[J\left(z\right)\right]f_{\mathrm{BE}}\left(\beta \zeta_{i}\right)e^{-i\zeta_{i}t}-2\pi i\sum_{j}{\mathrm{Res}}_{z=\xi_{j}}\left[f_{\mathrm{BE}}\left(z\right)\right]J\left(\xi_{j}/\beta \right)e^{-i\left(\xi_{j}/\beta \right)t}$$

where {ζ_*i*_} are the first-order poles in the lower half of the complex plane of J(ω) and {ξ_*i*_} are those of *f*_BE_(*βω*). By this method, the BCF can always be decomposed in terms of complex decaying exponentials such that *C*(*t*) = ∑_*k*=1_^*K*^ *c*_*k*_ *e* ^γ_*k*_ *t*^ and C*­(*t*) = ∑_*k*=1_^*K*^ *c̅*_*k*_ *e* ^γ_*k*_ *t*^, where *c*_*k*_, *c̅*_*k*_, and γ_*k*_ are complex numbers. Each term *k* in eq 4 corresponds to a feature and *K* is the total number of features.^17^ The first set of features arises due to the spectral density itself, while the second set arises due to the thermal factor and is referred to as low-temperature corrections.

Two common models for the spectral density are the Drude–Lorentz (DL) model and the Brownian oscillator (BO) model.^19,50^ The DL spectral density,

5 $$J_{\mathrm{DL}}\left(\omega \right)=\frac{2\lambda }{\pi }\frac{\omega_{c}\omega }{\omega^{2}+\omega_{c}^{2}}$$

describes an Ohmic environment with λ reorganization energy and ω_c_ cutoff frequency. Drude–Lorentz environments contribute to a single, simple decaying exponential with a characteristic relaxation time scale of ω_c_^–1^. In turn, the BO spectral density is expressed as

6 $$J_{\mathrm{BO}}\left(\omega \right)=\frac{4\lambda }{\pi }\frac{\eta \omega_{0}^{2}\omega }{{\left(\omega^{2}-\omega_{0}^{2}\right)}^{2}+4\eta^{2}\omega^{2}}$$

and describes the contribution of a discrete harmonic oscillator, where λ is its reorganization energy, ω_0_ its natural frequency, and 1/η its damping rate. Brownian oscillators contribute two oscillatory-decaying features to the BCF decomposition. In TENSO, the actual spectral density used for the BO oscillator is

7 $$J_{\mathrm{BO}}\left(\omega \right)=\frac{4\lambda \eta }{\pi }\frac{({\omega_{0}}^{\prime 2}+\eta^{2})\omega }{[{\left(\omega +{\omega_{0}}^{\prime }\right)}^{2}+\eta^{2}]\left[{\left(\omega -{\omega_{0}}^{\prime }\right)}^{2}+\eta^{2}\right]}$$

which is mathematically equivalent to the standard Brownian oscillator in eq 6 under the parameter mapping ${\omega_{0}}^{\prime }=\sqrt{\omega_{0}^{2}-\eta^{2}}$ , which corresponds to the effective frequency under damping (η < ω_0_). The factored form is used because its relevant poles are simply located at ± ω_0_^′^ – *iη*, which makes the decomposition in eq 4 straightforward. In chemical dynamics, it is often possible to define a spectral density as a combination of DL and BO terms as^10^

8 $$J\left(\omega \right)=J_{\mathrm{DL}}\left(\omega \right)+\sum_{b}J_{\mathrm{BO}}^{\left(b\right)}\left(\omega \right)$$

Environments with a large number of features, *K*, are termed highly structured. Since the numerical cost of the open quantum dynamics scales exponentially with *K*, the computations become increasingly challenging. In fact, HEOM calculations using standard methods are only feasible with one to five features. By contrast, in TENSO, the spectral density that describes the thermal environment can be highly structured as needed to describe baths of chemical complexity. However, the environment itself must be harmonic or, alternatively, mapped to a surrogate harmonic bath, which has been shown to serve as an accurate model of condensed-phase environments.^73^

### 2.2 Hierarchical Equations of Motion

While the full dynamics of the composite system with density matrix ρ­(*t*) is unitary, the dynamics of the reduced system, ρ_S_ = Tr_B_(ρ­(*t*)), is nonunitary, where Tr_B_ indicates a trace over the bath degrees of freedom. The dynamical map of ρ_S_(*t*) is^74^

9 $${\mathrm{\rho ̃}}_{S}\left(t\right)=T\mathrm{F̃}\left(t,0\right)\rho_{S}\left(0\right)$$

with time-ordering operator T and

10 $$\mathrm{F̃}\left(t,0\right)=e^{-\int_{0}^{t}\mathrm{ds}{\mathrm{Q̃}}_{S}^{\times }\left(s\right)\int_{0}^{s}\mathrm{du}{\left(C\left(s-u\right){\mathrm{Q̃}}_{S}\left(u\right)\right)}^{\times }}$$

Throughout, the tilde indicates operators in the interaction picture of *H*_0_(*t*) = *H*_S_(*t*) + *H*_B_,

11 $$Õ\left(t\right)={\left(Te^{-i\int_{0}^{t}H_{0}\left(t\prime \right)\mathrm{dt}\prime }\right)}^{†}O\left(t\right)\left(Te^{-i\int_{0}^{t}H_{0}\left(t\prime \right)\mathrm{dt}\prime }\right)$$

For any operator *A*, we use the convention *A* ^×^ *B* = *A* ^>^ *B* – *A* ^<^ *B* = *AB* – *BA* ^†^.

Employing the decomposition of the structured bath into features following eq 4 and calculating the time derivatives in the formal evolution of ρ̃_S_(*t*) reveals that the influence of the thermal environment is exactly captured by an infinite hierarchy of auxiliary density matrices (ADMs), {*ϱ*_*n⃗*_(*t*)}, of the same dimension, *M* × *M*, as the system density matrix. Here *n⃗* = (*n*_1_, ...*n*_*k*_, ...*n*_*K*_) is a *K*-dimensional index, with *K* being the number of features of the bath. Each *n*_*k*_ runs formally from 0 to infinity and practically from 0 to a truncation limit known as the hierarchy depth.

TENSO takes the generalized bexcitonic view of HEOM,^17^ where the system is conceptualized as interacting with a collection of fictitious, bosonic quasiparticles called bexcitons, each corresponding to a bath feature. In the bexcitonic HEOM, the ADMs are arranged into an extended density operator (EDO). The EDO is |Ω­(*t*)⟩ = ∑_*n⃗*_ *ϱ*_*n⃗*_(*t*)|*n⃗*⟩, which is specified in the basis given by |*n⃗* ⟩ = |*n*_1_⟩⊗···⊗ |*n*_*k*_⟩⊗··· ⊗ |*n*_*K*_⟩ such that a given ADM is found as *ϱ*_*n⃗*_(*t*) = ⟨*n⃗*|Ω­(*t*)⟩. The system density matrix is ρ_S_(*t*) = *ϱ*_*0⃗*_(*t*) = ⟨0⃗|Ω­(*t*)⟩, meaning it is found where all indices in |*n⃗*⟩ are zero.

The equation of motion of the EDO is given by^14,17^

12 $$\frac{d}{\mathrm{dt}}\left|\Omega \left(t\right)\right\rangle =\left(-iH_{S}^{\times }\left(t\right)+\sum_{k=1}^{K}D_{k}\right)\left|\Omega \left(t\right)\right\rangle$$

where $D_{k}$ is the dissipator associated with the *k*th bath feature, or bexciton. In particular, for each *k*,

13 $$D_{k}=\gamma_{k}{\mathrm{\alpha ̂}}_{k}^{†}{\mathrm{\alpha ̂}}_{k}+\left(c_{k}Q_{S}^{>}-{\mathrm{c̅}}_{k}Q_{S}^{<}\right){ẑ}_{k}^{-1}{\mathrm{\alpha ̂}}_{k}^{†}-Q_{S}^{\times }{\mathrm{\alpha ̂}}_{k}{ẑ}_{k}$$

with *ẑ*_*k*_, the metric, being any invertible operator which commutes with α̂_*k*_^†^α_*k*_, α̂_*k*_^†^ and α̂_*k*_ are the bosonic creation and annihilation operators for the *k*th bexciton, ([α̂_*k*_, α̂_*k*′_^†^]) = δ_*k*,*k*′_. In the bexcitonic view of HEOM, each feature, *k*, is associated with a fictitious, bosonic quasiparticle. The operator α̂_*k*_^†^ creates a *k*th bexciton and the operator α̂_*k*_ destroys it. With |*n⃗*⟩, we associate a state with |*n*_*k*_⟩ bexcitons created for each *k*. The *K* bexcitons constitute a coarse-grained, but numerically exact, representation of the macroscopic bath but are not physical particles associated with modes of the bath. Although useful for monitoring convergence of the dynamics, bexcitons do not provide physical information about the bath state.

For each feature *k*, the bexcitonic ladder must be truncated at a maximum occupation (*N*_*k*_ – 1), the depth of the *k*th-bexciton, to allow for practical simulations.^17^ For an *M*-state system coupled to *K* bath modes, each truncated at $N_{k}=O\left(N\right)$ , the memory cost of the EDO scales as $O\left(M^{2}N^{K}\right)$ . This exponential growth of memory requirements with *K* constitutes the principal limitation of HEOM simulations.^14^ This problem is especially severe for highly structured spectral densities and at low temperatures, where many features are required.

Equation 12 specifies a class of QMEs. Conventional HEOM uses *ẑ*_*k*_ = i­(α̂_*k*_^†^α̂_*k*_)^−1/2^ and a number basis representation of |*n⃗*⟩. By adjusting the metric and basis representation of |*n⃗*⟩, we can recover and develop many variants of HEOM.^17^

### 2.3 Tree Tensor Network Decomposition

The curse of dimensionality in HEOM can be mitigated by recognizing that the EDO contains significant redundancy that can be systematically compressed via tensor network representations, greatly reducing memory requirements.^14,75–77^ We begin by reexpressing the EDO. Analogously to the density matrix of the system, ρ_S_(*t*), with matrix elements [ρ_S_]_*ij*_ = ⟨*i*|ρ_S_(*t*)|*j*⟩ in a basis {|*i*⟩} spanning the system Hilbert space, the EDO can be represented as

14 $${\left[\Omega \left(t\right)\right]}_{\mathrm{ij}n_{1}\cdot \cdot \cdot n_{K}}=\left\langle i\right|\left\langle n_{1}\cdot \cdot \cdot n_{K}\right|\Omega \left(t\right)\rangle \left|j\right\rangle$$

where {|*n*_*k*_⟩}_*n*_*k*_=0_^*N*_*k*_–1^ is the truncated number basis for the *k*th-bexciton. The dynamics of Ω­(*t*) in eq 12 can be written compactly as^14^

15 $$\frac{d}{\mathrm{dt}}\Omega \left(t\right)=L\left(t\right)\Omega \left(t\right)$$

where L(t) is the tensor representation of the Liouvillian superoperator generating the dynamics in eq 12. Because the basis factorizes as |*i*⟩⊗ ⟨*j*|⊗ |*n*_1_⟩⊗ ···⊗ |*n*_*K*_⟩, L(t) admits the operator in sum-of-products (SoPs) form

16 $$L\left(t\right)\equiv \sum_{m=1}^{5K+2}h_{m}^{>}\left(t\right)⊗h_{m}^{<}\left(t\right)⊗h_{m}^{\left(1\right)}⊗\cdot \cdot \cdot ⊗h_{m}^{\left(K\right)}$$

with local operators *h*_*m*_^(κ)^ where the index *m* runs over all terms in eq 12 and (κ = >, <, 1, ..., *K*). Each dissipator $D_{k}$ contributes five terms and the unitary Liouvillian, −*iH*_*S*_^×^(*t*), contributes two. Here, *h*_*m*_^>^(*t*) and *h*_*m*_^<^(*t*) act on the system space while *h*_*m*_^(*k*)^ acts on the truncated *k*th-bexciton space.

The EDO is a high-dimensional tensor. The memory requirements to describe a tensor are exponential in the tensor’s order, meaning that for a tensor *A*_*a*_1_...*a*_*D*__, where the *a*_*i*_ = 1,···,*R*, the tensor requires *O*(*R* ^*D*^) memory space. Performing mathematical operations on the tensor that require access to all elements, thus also scales catastrophically. To avoid this, the EDO is compressed by expressing it as a network of low-order core tensors. The tree is assembled in general by repeated applications of the singular value decomposition^78^ (SVD), a procedure known as the hierarchical Tucker decomposition (HTD).^79,80^ When performing the decomposition, small singular values judged to be relatively unimportant are discarded, with the number of retained singular values known as the rank of the bond, *R*. Contractions are performed between the network’s core tensors according to the topology of a tree graph, and the lowest-order tensors possible are employed for efficiency. The lowest order that allows for the assembly of an arbitrary-sized TTN is three. This decomposes *K* order-three core tensors with *K* – 1 contractions.

The simplest TTN topology is a tensor train (TT),

17 $$\Omega_{\mathrm{ij}n_{1}\cdot \cdot \cdot n_{K}}=\sum_{a_{1}a_{2}\cdot \cdot \cdot a_{K-1}}^{R_{1}R_{2}\cdot \cdot \cdot R_{K-1}}A_{\mathrm{ij}a_{1}}^{\left(0\right)}U_{a_{1}n_{1}a_{2}}^{\left(1\right)}U_{a_{2}n_{2}a_{3}}^{\left(2\right)}\cdot \cdot \cdot U_{a_{K-1}n_{K-1}n_{K}}^{\left(K-1\right)}$$

where {*a*_*s*_}_*s*=1_^*K*–1^ are bond indices of ranks {*R*_*s*_}. Similarly, a general TTN is written as

18 $$\begin{array}{l} {\left[\Omega \left(t\right)\right]}_{\mathrm{ij}n_{1}\cdot \cdot \cdot n_{K}}=\sum_{a_{1}\cdot \cdot \cdot a_{K-1}}^{R_{1}\cdot \cdot \cdot R_{K-1}}A_{\mathrm{ij}a_{1}}^{\left(0\right)}U_{a_{1}\beta_{1}\gamma_{1}}^{\left(1\right)}\cdot \cdot \cdot U_{a_{K-1}\beta_{K-1}\gamma_{K-1}}^{\left(K-1\right)}\\ \equiv {\left[\mathrm{Con}\left(A^{\left(0\right)}\left(t\right),U^{\left(1\right)}\left(t\right),...,U^{\left(K-1\right)}\left(t\right)\right)\right]}_{\mathrm{ij}n_{1}\cdot \cdot \cdot n_{K}} \end{array}$$

where Con(·) denotes contraction of the TTN core tensors, *A* ^(0)^(*t*), *U* ^(1)^(*t*), ...*U* ^(*K*–1)^(*t*), according to the chosen network topology, and each pair (β_*s*_,γ_*s*_) corresponds either to a bath index *n*_*k*_ (open bond) or to a virtual bond *a*_*u*_ (internal contraction).

The semiunitary core tensors *U* ^(*s*)^(*t*) in the TTN are chosen to satisfy

19 $$\sum_{\beta \gamma }{\left[U^{\left(s\right)}\left(t\right)\right]}_{{a^{\prime }}_{s},\beta \gamma }^{*}{\left[U^{\left(s\right)}\left(t\right)\right]}_{a_{s},\beta \gamma }=\delta_{{a^{\prime }}_{s}a_{s}}$$

but the root tensor *A* ^(0)^(*t*) is not constrained to be semiunitary. Following standard practice, the system indices (*i*,*j*) and the first bond *a*_1_ are placed in the root tensor so that the physical system degrees of freedom remain uncompressed while the bath degrees of freedom are compacted.

For a hierarchy of depth *N* and TTN bond ranks *R*_s_ = *O*(*R*), the TTN storage scales as

20 $$O\left(M^{2}R+\mathrm{KNR}\left(N+R\right)\right)$$

thereby eliminating the exponential dependence on *K* and providing a controllable, systematically improvable approximation that becomes exact as the ranks increase. The equation of motion for the tensor network is determined by the TDVP applied using the Liouvillian equation, eq 15. The derivation is detailed in ref 14 with the equations of motion for order three tensors in eqs 19 and 20, and their generalization to a TTN containing tensors with arbitrary order in eqs S14 and S15 of its supplementary information. In practice, the bond dimension or rank *R* must be increased until convergence. The smaller the rank that can be used, the more efficient the compression of the TTN. To produce numerically exact results, TENSO also requires a converged hierarchy depth, low integration error, and a sufficiently accurate decomposition of the BCF as needed by any HEOM-based computation.

The most important result is the determination of equations of motion for the root and core tensors, admitting general numerical methods for solving coupled differential equations, for any master equation that admits a sum-of-products form for L(t) . This allows flexibility in the implementation of propagation methods, including both fixed rank and adaptive rank integration schemes.

TENSO implements three different propagation strategies: direct and two versions of projector-splitting. The direct integration strategy, known as vmf in the package, is a fixed rank method that simultaneously integrates the nonlinear coupled series of ordinary differential equations representing the evolution of the core and root tensors. These equations of motion involve inversion of matrices that are singular during the early steps of the dynamics, leading to the need for regularization techniques and, hence, regularization errors. This propagation strategy and the high-order regularization technique implemented in TENSO are detailed in Section IID1 in ref 14. The advantage of this strategy is that it enables coupling the TTN-HEOM to well-developed solvers of ordinary differential equations based on Runge–Kutta and other schemes that allow for larger integration time steps, adaptive time steps, and even parallelization.

By contrast, the projector-splitting methods propagate the tensors in the network individually and sequentially based on Trotterization of the propagator. This approach avoids the regularization but is limited by the Trotterization errors, which require small time steps. At each step of the projector-splitting methods, the root tensor is moved through a complete traversal of the tree, passing to an adjacent semiunitary tensor via SVD at each substep. In this way, every core tensor is propagated while it is the root, for which the dynamics does not have a singularity issue. The two algorithms, ps1 and ps2, differ in that ps1 moves tensors to the root during propagation sweeps without adjusting the rank. In turn, ps2 merges neighboring tensors as it moves them to the root and performs an SVD to split the combined tensor back into two, resulting in an adaptive rank algorithm. For detailed algorithms that are implemented in TENSO, see Section IID in ref 14.

## 3 Numerical Examples

### 3.1 Obtaining and Installing TENSO

TENSO is a Python package that can be obtained from github: https://github.com/ifgroup/pytenso. Users must have Python 3.13 or later installed to run TENSO. TENSO further relies on the packages numpy,^81^ scipy,^82^ pytorch,^83^ torchdiffeq^84^ and tqdm.^85^ These packages provide high-level interfaces designed to simplify tensor operations across diverse computational platforms, including various CPU and GPU architectures. For this tutorial, users should also have matplotlib^86^ available. A Python virtual environment should be prepared for TENSO, and these packages should be made available in it prior to installation. A thorough guide to preparing virtual environments and installing Python packages for Windows, Macintosh, and Linux users can be found in the official Python 3 documentation. Package managers Anaconda and Pip can also be used. Once the environment has been prepared and TENSO installed, run it as python <input file>. TENSO reads all parameters of the system and bath from the input file using energy units of cm^–1^, time units of fs, and temperature units of K.

### 3.2 Overview

In this section, we will use three sample systems, the spin-boson, the FMO complex, and a pair of qubits, all selected due to their important roles in quantum dynamics, to demonstrate how to employ TENSO on research problems ranging from energy transport to entanglement dynamics.

There are four main steps to running a TENSO calculation: First, the libraries and modules are imported. Second, the bath correlation function decomposition is generated by the function gen_bcf or provided manually by an advanced user. Third, the initial system state, system-bath coupling, tensor network structure, and propagator are initialized by the function system_multibath. Finally, propagation is carried out to the desired final time using the tqdm package to display progress and estimate the time remaining.

All examples in this tutorial are straightforward to run on a laptop or desktop computer, with the exception of the Fenna-Matthews-Olson complex example, which is computationally more demanding. TENSO excels at and provides the largest performance gains when handling difficult problems for HEOM, those which have complicated spectral densities that cannot be represented by a single Drude–Lorentz or Brownian bath alone, or those which need large hierarchy depths.

### 3.3 Example 1: Spin-Boson

The spin-boson model, a single two-level system interacting with a thermal boson bath,^19,49^ has been used to test the performance of quantum simulation methods, Markovian and non-Markovian,^87–90^ to study the impacts of bath parameters in open systems,^91,92^ and to understand physical processes involving two-level systems (TLS), ranging from spontaneous emission^49,93^ to superconducting qubit relaxation.^94,95^ It remains an important system of study.

The spin-boson system Hamiltonian is given by

21 $$H_{S}=\frac{\Delta \epsilon }{2}\sigma_{z}+V\sigma_{x}$$

with energy gap Δϵ, tunneling amplitude *V*, and Pauli operators σ_*z*_ and σ_*x*_. The system-bath coupling is

22 $$H_{\mathrm{SB}}=\frac{\sigma_{z}}{2}⊗X_{B}$$

We will use the spin-boson problem to demonstrate the basic usage of TENSO, how to specify the spectral density of a structured bath, how to invoke a time-dependent Hamiltonian, and how to understand parameters that may impact numerical convergence of the results.

#### 3.3.1 Dynamics in a Structured Bath

Listing 1 shows a complete TENSO input file specifying an HEOM calculation for a spin in interaction with a bath whose spectral density includes a DL and a BO term. The file is broken into four sections: (1) importing modules, (2) bath correlation function definition, (3) propagator setup, and (4) propagation. All HEOM examples in this tutorial will require the same import statements. Simulation parameters used in Listing 1 are given in Table 1.

**Table 1:** Simulation Parameters for the Spin–Boson Model^a^

parameter	value (cm^–1^)
Δϵ	1500
*V*	300
λ, ω_c_ (DL)	540, 70
λ, η, ω_0_^′^ (BO)	161.6, 10, 1243

a The system parameters include the energy bias *Δϵ* and the tunneling coupling *V*. The bath spectral density is modeled as a sum of Drude–Lorentz (DL) and Brownian oscillator (BO) contributions, parametrized by the reorganization energy λ, the cutoff frequency ω_c_, the damping coefficient η, and the vibrational frequency ω_0_^′^. The resulting spectral density is shown in Figure 1b. All parameters are given in cm^–1^.

In TENSO, the gen_bcf helper function builds the BCF. It takes the cutoff frequencies, the reorganization energies, widths, and the resonant frequencies as lists that encode the spectral densities’ structures. To label the two types of SDs, TENSO uses the suffixes _d and _b for the DL and Brownian SD, respectively. To use the Matsubara decomposition scheme, the argument “Pade” is replaced with “Matsubara” in the call to gen_bcf. It is also possible to implement custom BCFs and their decompositions, as detailed in Section 7.

Construction of the propagator in TENSO is performed with the system_multibath function. The bath correlation object produced by gen_bcf is needed by system_multibath to construct the propagator. The system_multibath helper function can address one or more baths. It takes all system information, the BCF or BCF’s in list form, system-bath coupling operators, and all additional simulation parameters such as integrator tolerances or the tensor network decomposition scheme. The tqdm package then uses the propagator to advance the system through time and displays a status bar of the calculation’s progress.
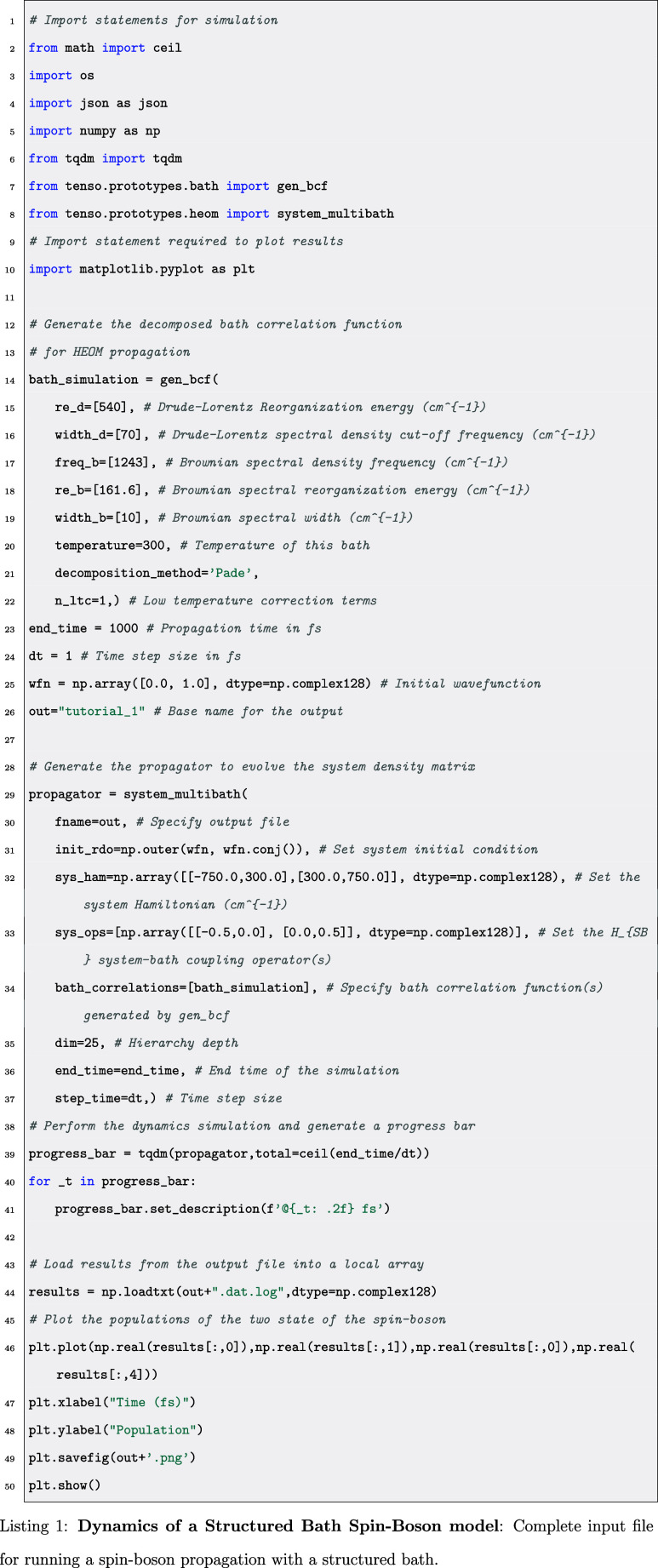


On completion, this calculation will display a figure of the populations of the two states and save the image to a file. The output file, tutorial_1.dat.log will record the complex values of all entries in the density matrix of the evolving system and output them in the order ρ_1,1_, ρ_1,2_... ρ_*n*,*n*–1_, ρ_*n,n*_ for each simulation time step. The dynamics are shown in Figure 1.

**Figure 1:**
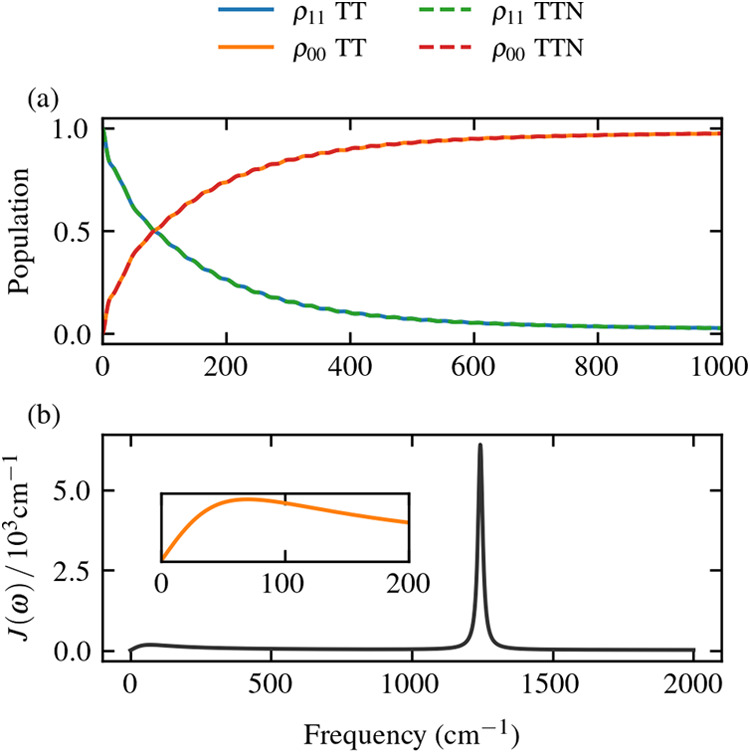
Structured spin-boson quantum dynamics. (a) Population relaxation dynamics of the spin-boson system, with the excited state in blue and ground state in orange, showing that the dynamics are identical whether the TT or BTT tensor network decomposition is employed. (b) Structured spectral density employed in the simulation.

#### 3.3.2 Comparison of Different Tensor Decompositions

TENSO incorporates two built-in TTN decomposition schemes, the tensor train (TT) decomposition and the balanced tensor tree (BTT) decomposition. The binary BTT is the default option and will be appropriate for most uses. The first example in Listing 1 addressed a spin-boson system using the BTT. We now demonstrate that a TT decomposition produces equivalent results. To produce an equivalent calculation with a TT decomposition, we add the line frame_method= “train”, in Listing 1 as an extra argument to system_multibath. This is the only modification needed.

Figure 1 shows results of both the TT and BTT simulations of the spin-boson problem, demonstrating that they produce equivalent results. The dynamics show decay of the population and small oscillations attributed to interaction with the underdamped Brownian mode, whose presence is very notable in the spectral density, Figure 1b. Note that all of TENSO’s convergence parameters controlling accuracy and expense, which will be discussed in depth in Section 3.3.6, are identical for these calculations. In this particular case, the convergence and performance of the simulation are not significantly impacted by the chosen tensor configuration.

#### 3.3.3 Noncommuting Fluctuations

TENSO, as it is an HEOM-based method, enables modeling of open quantum systems coupled to noncommuting fluctuations. In this situation, an open quantum system is coupled to two or more distinct, independent thermal baths. Specifically, with the superscript b referencing distinct baths, *H*_SB_ = ∑_b_ *Q*_S_^(b)^⊗*X*_B_^(b)^, $H_{B}=\sum_{j,b}\left(\frac{{\left(p_{j}^{\left(b\right)}\right)}^{2}}{2m_{j}^{\left(b\right)}}+\frac{m_{j}^{\left(b\right)}{\left(\omega_{j}^{\left(b\right)}\right)}^{2}{\left(x_{j}^{\left(b\right)}\right)}^{2}}{2}\right)$ , and the *Q*_S_^(b)^ do not commute. These systems may display interesting phenomena, including the apparent suppression of spin relaxation by decoherence^96,97^ but their treatment can be challenging.^96–99^ As many systems relevant to quantum information science involve noncommuting fluctuations, including systems interacting simultaneously with radiation and phonon fields, addressing them is an important capability of TENSO.

A simple example of a system coupled to noncommuting fluctuations is a spin-boson problem where H_SB_ = $\frac{1}{2}\sigma_{z}⊗X_{B}^{\left(1\right)}+\frac{1}{2}\sigma_{x}⊗X_{\left(B\right)}^{\left(2\right)}$ . We calculate the dynamics of the system specified in Listing 1 and in Table 1 with the addition of a second, identical bath with $Q_{S}^{\left(2\right)}=\frac{1}{2}\sigma_{x}$ . This requires changing the propagator as shown in Listing 2.
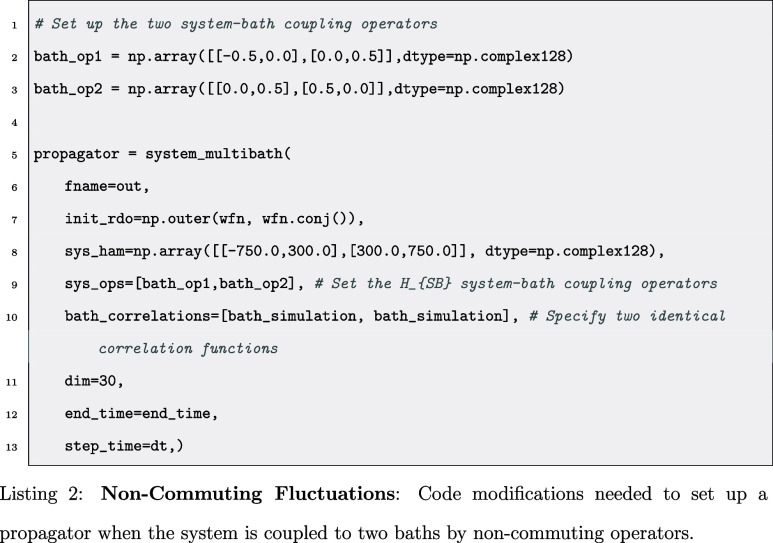


The dynamics of the system coupled to the two noncommuting fluctuations, shown in Figure 2, differ significantly from those of the system coupled to a single bath in Figure 1. The total reorganization energy has doubled with the inclusion of the second bath, leading to a faster relaxation. The apparent slowing of relaxation at approximately 50 fs appears to be due to the competing influences of the two baths interacting with the system.

**Figure 2:**
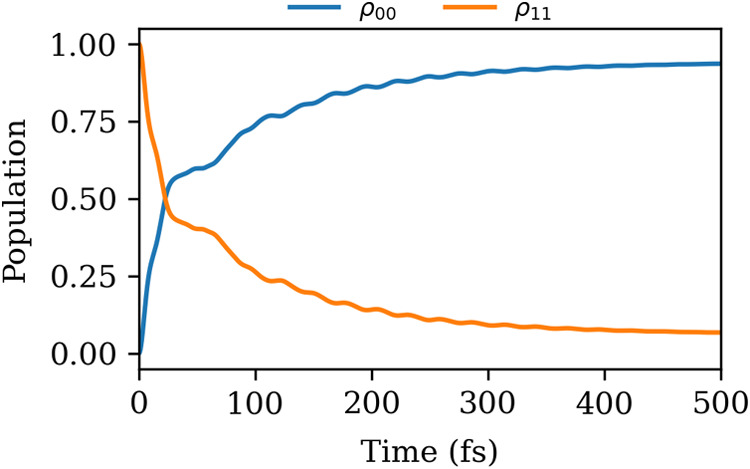
Spin-boson with two baths: evolution of a spin-boson system coupled to two noncommuting fluctuations.

#### 3.3.4 Driven Structured Spin-Boson Model

TENSO supports time-dependent Hamiltonians, enabling the simulation of driven phenomena in the presence of decoherence, such as photoexcitation of molecules in the condensed phase, spectroscopy, quantum control, and quantum information processing.

Here, we simulate a spin-boson model resonantly driven by a $\frac{\pi }{2}$ -pulse. In the absence of decoherence, this control pulse implements a perfect Hadamard gate on this qubit, transitioning it from the ground state |0⟩ to $\frac{1}{\sqrt{2}}\left(\left|0\right\rangle +\left|1\right\rangle \right)$ . The total system Hamiltonian is given by

23 $$H_{S}=\sigma_{z}\frac{\Delta \epsilon }{2}-\sigma_{x}\mu_{0}E\left(t\right)$$

where μ_0_ is the transition dipole moment and *E*(*t*) is the time-dependent laser field. The driving is realized by a single π/2-area Gaussian laser pulse, determined from the area theorem,^100^

24 $$E\left(t\right)=E_{0}\;\exp\left[-\frac{1}{2}{\left(\frac{t-\tau }{\sigma }\right)}^{2}\right]\;\cos\left(\omega_{L}t\right)$$

where *E*_0_ is the peak electric field amplitude, ω_*L*_ is the driving frequency, and σ is related to the pulse full width at half-maximum (FWHM) by $\sigma =\mathrm{FWHM}/\left(2\sqrt{2\;\ln2}\right)$ . In our simulations, σ corresponds to a value of 2.1233045 fs. The system-bath coupling corresponds to pure dephasing noise, which is appropriate in many quantum computing investigations where dephasing is often much faster than dissipation.

This simulation is carried out in TENSO by defining the laser parameters and a function laser_field_hadamard­(t) which returns the value of −μ_0_ *E*(*t*) with a maximum value of 3133.625 cm^–1^. The parameters for the bath are the same as those for the previous example outlined in Table 1, and the BCF portion of the input file requires no changes. The propagator must be modified to specify the time-dependent function and its associated operator, σ_*x*_. The necessary changes to the previous example code are shown in Listing 3.
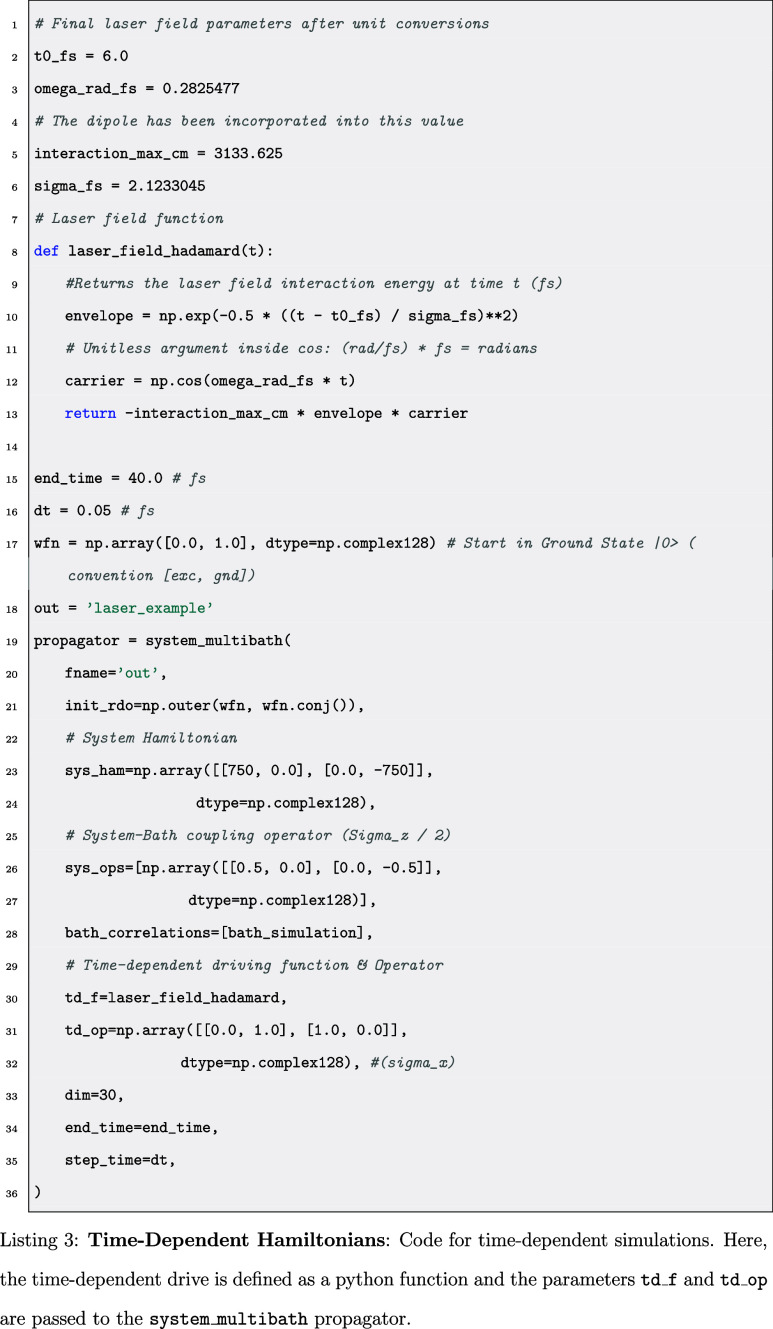


In Figure 3, the system’s state is driven to a superposition; however, this half-rotation in the Bloch sphere is not complete. The system state never reaches $\frac{1}{\sqrt{2}}\left(\left|0\right\rangle +\left|1\right\rangle \right)$ and the coherences decay quickly due to dephasing, meaning that a noisy Hadamard gate has been modeled. This demonstrates TENSO’s capability to simulate the impact of structured environmental noise on driven quantum systems, a situation relevant to many quantum control and quantum computing problems.

**Figure 3:**
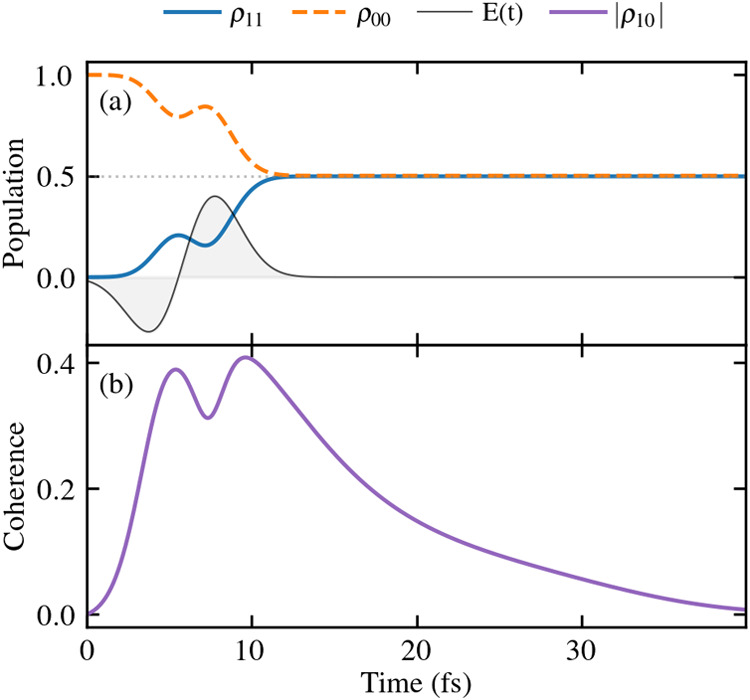
Driven spin-boson dynamics: (a) Time evolution of populations and (b) coherence evolution under a resonant π/2 Gaussian pulse while interacting with a structured bath. (c) The structured spectral density of the thermal bath. The laser-induced control dynamics demonstrates coherent excitation in the two-level system.

#### 3.3.5 Adaptability of the Framework to Other Equations of Motion

Although the principal application of TENSO and the principal focus of this tutorial is HEOM, the tensor network framework underlying the program is easily adapted to other equations of motion with propagators in sum-of-products form. In particular, this is demonstrated by the implementation of the thermofield strategy using ML-MCTDH, which is a tensor-based method for solving the time-dependent Schrödinger equation, in TENSO.^64^ The thermofield strategy allows the wave-function-based MCTDH to simulate finite-temperature dynamics by mapping them to zero-temperature dynamics in an extended Hilbert space.^39,101,102^ Due to the multilayered tree structure, the MCTDH implementation constitutes the ML-MCTDH method.

Performing an MCTDH calculation requires discretizing the bath, and the situation in Figure 1 requires a large discretization and significant computational resources. The technical aspects of bath discretization, as required to use MCTDH for thermal dynamics using TEDOPA and related methods, are detailed in refs 64, 39, 103, and 104. For the purposes of this demonstration, a system with a single Brownian spectral density that does not require as many computational resources to achieve MCTDH convergence has been selected to compare to HEOM. The required changes to the simulation code to perform MCTDH are demonstrated in Listing 4. The HEOM modules are replaced with MCTDH modules, and gen_bcf is replaced by gen_star_boson to perform the MCTDH discretized bath decomposition. The overall changes required to perform the simulation are minimal, demonstrating the versatility of the TENSO’s implementation framework and usage. The HEOM comparison for this example is generated by adjusting Listing 1 so that the bath contains only the Brownian mode specified in Listing 4. MCTDH calculations and their efficiency in comparison to HEOM calculations in TENSO’s tensor network approach are largely beyond the scope of this tutorial, and interested readers may find an in-depth discussion in ref 64.

Despite our focus on HEOM rather than MCTDH, we would be remiss not to offer a general comment on convergence. The discretization of the bath in MCTDH inevitably leads to recurrence and inaccuracy after a finite propagation time, which can be mitigated by increasing n_discretization but cannot be escaped entirely. In Figure 4, the selected discretization is sufficient for 200 fs but not for 300 fs.
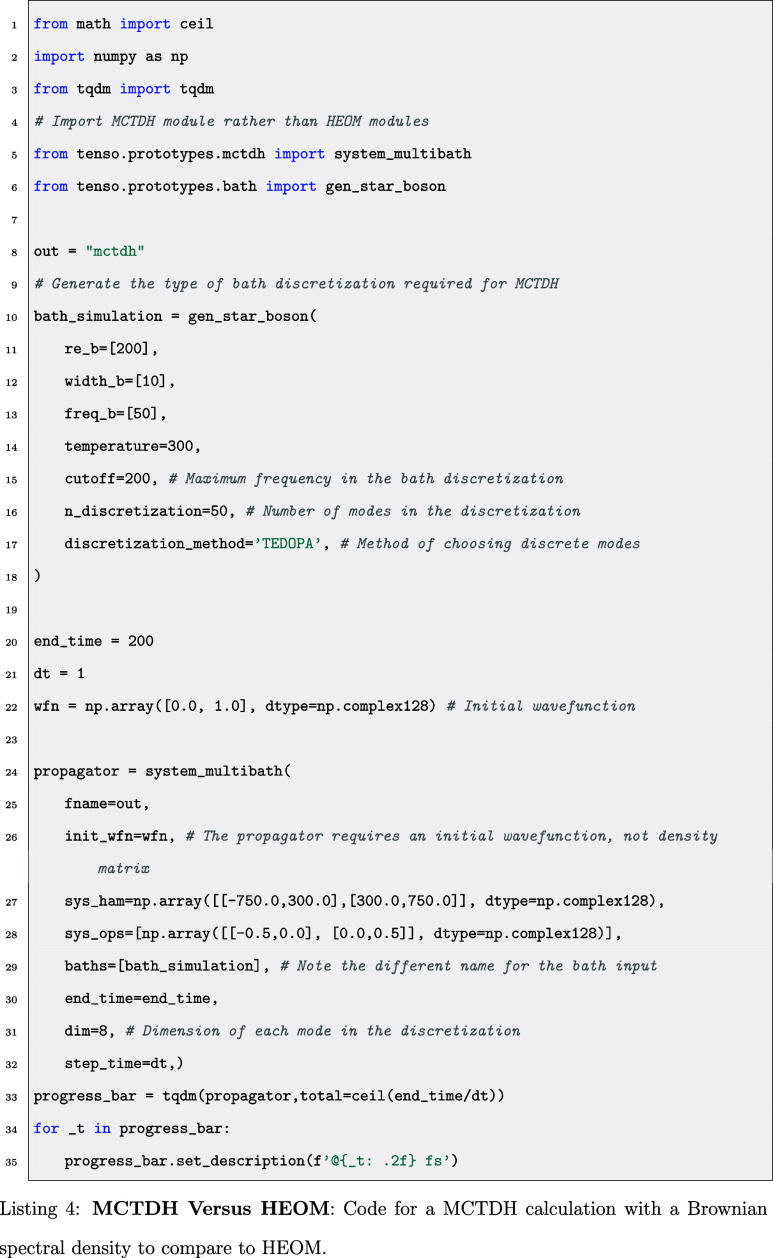


**Figure 4:**
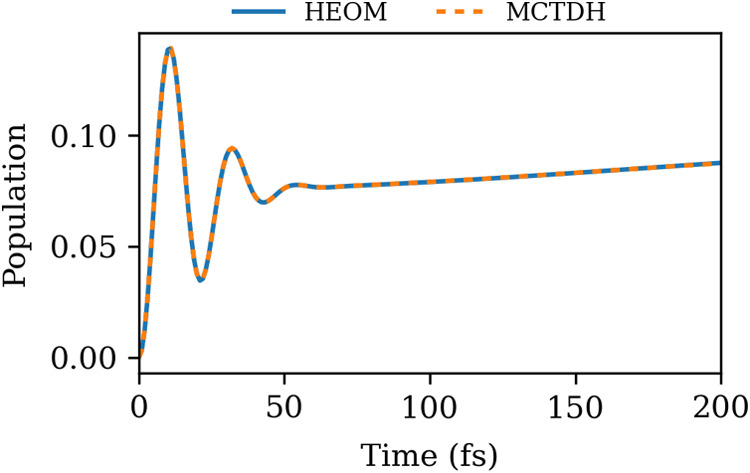
MCTDH vs HEOM. Short comparison of an MCTDH and HEOM calculation for a simple Brownian bath at 300 K. The HEOM calculation was carried out with n_ltc = 2, dim = 20, and dt = 1 with the default mixed propagation strategy.

#### 3.3.6 Convergence Parameters

TENSO has several parameters that control the numerical accuracy of the propagation, including the time step size, propagation method, rank of the tensor network, hierarchy depth, and number of low-temperature corrections included in the BCF decomposition. An increase in computational accuracy inevitably brings a higher computational cost.

The simplest adjustment parameter is the time step size. An initial step size that is too large may result in numerical problems or simulation failure. The initial step size is set by the parameter step_time in the propagator. A good rule of thumb is that it should be at most 1/20th of the fastest time scale in the problem.

The default propagation method in TENSO begins with the use of the ps2 projection splitting method that adapts the TTN ranks to the problem at hand and, therefore, has a variable memory footprint. TENSO then transitions to a static rank, constant memory footprint direct integration method, vmf. This scheme is appropriate for most uses. More information on adjusting the propagation method and error tolerances is located in Sections 4 and 7.5.

We will demonstrate convergence of calculations with respect to depth and rank using spin-boson models specified in Table 2. The first model in Figure 5a employs a symmetric model (Δϵ = 0) with a purely DL bath, while that in Figure 5b,c uses an asymmetric system coupled to a combined DL and BO bath. The system Hamiltonian and system-bath coupling operator are the same as in Listing 1.

**Figure 5:**
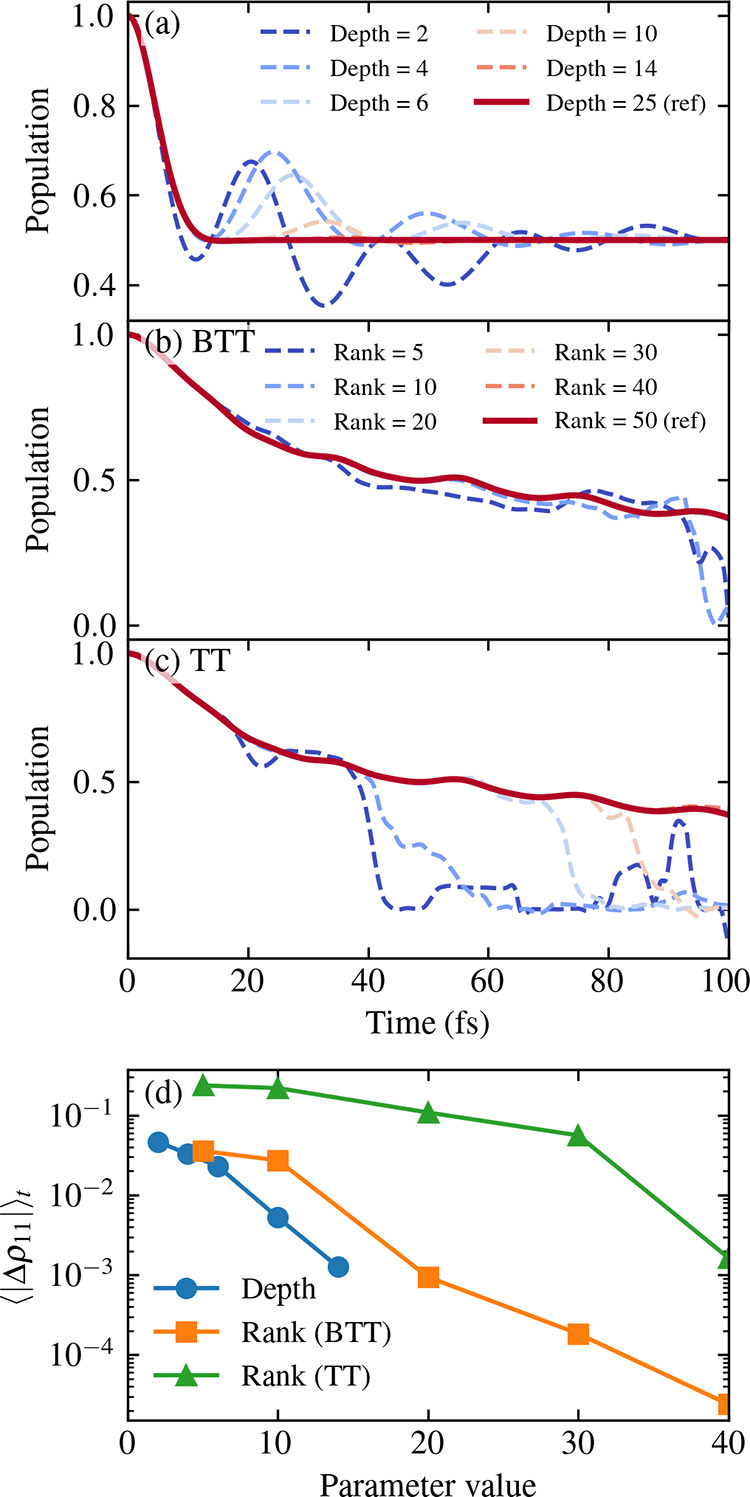
Convergence demonstration. (a) Population of a spin-boson model simulated with the BTT method for several different hierarchy depths demonstrating the spurious oscillations that occur when the depth is insufficient. (b) Population of a spin-boson model simulated with the BTT method for several different ranks, showing less sensitivity to rank. (c) Population of a spin-boson model simulated with the TT method for several different ranks showing more sensitivity to rank. (d) Comparison of mean absolute deviation of population from the reference population with depth and rank.

**Table 2:** Simulation Parameters for the Spin–Boson Models Used in the Convergence Tests of Figure 5 ^a^

parameter	panel a (cm^–1^)	panels b and c (cm^–1^)
Δϵ	0	1500
*V*	300	300
λ, ω_c_ (DL)	540, 70	540, 70
λ, η, ω_0_^′^ (BO)	0, 0, 0	330, 4, 1663

a All parameters are given in cm^–1^.

Truncation depth of the hierarchy expansion for each feature is controlled by the parameter dim in the propagator specifications. The default value is 5, but different models will require different depths. HEOM, when performed with an inadequate hierarchy depth, may produce dynamics with spurious oscillations or revivals^30,105^ so it is crucial to check convergence with respect to hierarchy depth.^72^ This issue is demonstrated in Figure 5a, which performs the same calculations as in Listing 1 with a few modifications to the parameters. Only the DL term is used in the spectral density, Δϵ = 0, and the system-bath coupling operator is *H*_SB_= σ_*x*_⊗*X*_*B*_. The truncation depth is set to dim = n for each of *n* = 2, 4, 6, 10, 14, 25. Oscillations in the population when *n* is less than 25, whether very dramatic at *n* = 2 or minimal at *n* = 14, are the result of insufficient hierarchy depth, but superficially resemble coherent dynamics that may occur in other quantum systems. Similar spurious oscillations can occur when insufficient low-temperature corrections are included,^72^ but this situation may produce not only spurious oscillations but also physically unreasonable dynamics such as negative populations.^106,107^ The number of low-temperature correction terms included during the bath decomposition is determined by n_ltc during BCF construction. The default parameter is 0. The number required depends on the specific system and bath energy scales. For example, in Listing 1, n_ltc = 1 is sufficient. Note that the HEOM requires increasingly more features in the environment as the temperature is lowered and can become numerically intractable.

The tensor network rank is also a critical convergence parameter governing the accuracy of the simulations. Figure 5b,c displays the convergence of the population dynamics with respect to the bond rank for the BTT and TT decompositions, respectively, in a case where *H*_SB_ = σ_*z*_⊗*X*_B_. The simulations use the fixed rank ps1 as the propagation strategy. The BTT structure in (b) exhibits quick convergence toward the reference solution (Rank = 50), whereas the TT decomposition in (c) requires significantly higher ranks to achieve comparable accuracy and displays unphysical artifacts at low ranks. The time-averaged absolute error versus the reference simulation, ${\left\langle |\Delta \rho_{11}|\right\rangle }_{t}=\frac{1}{N_{\mathrm{ts}}}\sum_{k=1}^{N_{\mathrm{ts}}}\left|\rho_{11}\left(t_{k}\right)-\rho_{11}^{\mathrm{ref}}\left(t_{k}\right)\right|$ , where *N*_ts_ is the number of time steps in the simulation, is shown in Figure 5d as a function of the parameter value for all three cases, providing a quantitative measure of convergence. The BTT rank converges most rapidly, reaching errors below 10^–3^ at moderate rank values, while the TT decomposition and hierarchy depth require larger parameter values to achieve similar accuracy.

Additional discussion of convergence testing is found in Section 4.

### 3.4 Example 2: FMO Complex with a Structured Environment

The Fenna–Matthews–Olson^51,52^ complex is employed in studies of excitonic energy transfer, coherence and entanglement in photosynthetic complexes, and for benchmarking new methods.^57,108–111^ Studies have examined non-Markovian effects and the impact of different thermal baths on the FMO complex to improve our understanding of photosynthetic processes.^53,55,112^ We will use the FMO complex to demonstrate TENSO’s utility in addressing larger systems with multiple baths and complicated descriptions of the environment’s spectral density involving three or more Brownian terms.

On the site basis, the FMO system Hamiltonian is

25 $$H_{S}=\sum_{i}\epsilon_{i}\left|i\right\rangle \left\langle i\right|+\sum_{i\neq j}V_{\mathrm{ij}}\left(\left|i\right\rangle \left\langle j\right|+\left|j\right\rangle \left\langle i\right|\right)$$

where ϵ_*i*_ denotes site energies and *V*_*ij*_ denotes the coupling between sites *i* and *j*. The FMO complex is usually described by seven or eight sites, but we will use a reduced three-site description to control computational cost. The three-site model captures major features effectively^113^ and will suffice for the purpose of our demonstration.

The matrix representation of the three-site Hamiltonian in cm^–1^ is^114^

$$H_{S}=\left[\begin{array}{lll} 200 & -87.7 & 5.5\\ -87.7 & 320 & 30.8\\ 5.5 & 30.8 & 0 \end{array}\right]$$

Most HEOM simulations of the FMO complex employ an independent bath with a Drude–Lorentz spectral density at each site. While this captures significant dynamical features, higher-frequency modes—which substantially influence short-time behavior—are typically underestimated;^115^ thus, simulations with more accurate spectral densities are desirable.^116,117^ TENSO makes studies incorporating structured spectral densities for this system computationally feasible for HEOM. To reduce the number of low-temperature corrections, high-frequency modes such as those encountered in the FMO model can be addressed by delta function treatment.^72^ This method is compatible with TENSO but is not currently implemented.

The system-bath coupling Hamiltonian is

26 $$H_{\mathrm{SB}}=\sum_{b}\sum_{i}\hbar \omega_{b,i}\sqrt{2S_{b,i}}\left|i\right\rangle \left\langle i\right|⊗X_{B}^{\left(b\right)}$$

with *S*_b,*i*_ a Huang–Rhys factor and ω_b,*i*_ the *i*th oscillator frequency of bath b. Note that each site is coupled to an independent bath. Our model approximates the spectral density via a sum of six underdamped Brownian oscillator terms, which approximate one recently determined from spectroscopic measurements.^118^

Listing 5 contains the entire input file necessary to run the FMO example, excluding the import statements, which are identical to Listing 1. Note that a different propagation method is used in this simulation. Propagators are discussed more in Sections 4 and 7.5. We will make additional small modifications to this input file to compare the FMO dynamics with different thermal baths. First, we will run calculations at both 300 and 77 K and use three low-temperature corrections, n_ltc = 3, for all 77 K calculations. Second, we will employ two simpler spectral densities, one using only the first three Brownian peaks, the three located below 1000 cm^–1^, and one using a simple Drude–Lorentz spectral density with λ^DL^ = 35 cm^–1^ and ω_*c*_^DL^ = 106.18 cm^–1^.^114^ These three spectral densities are shown in Figure 6c.
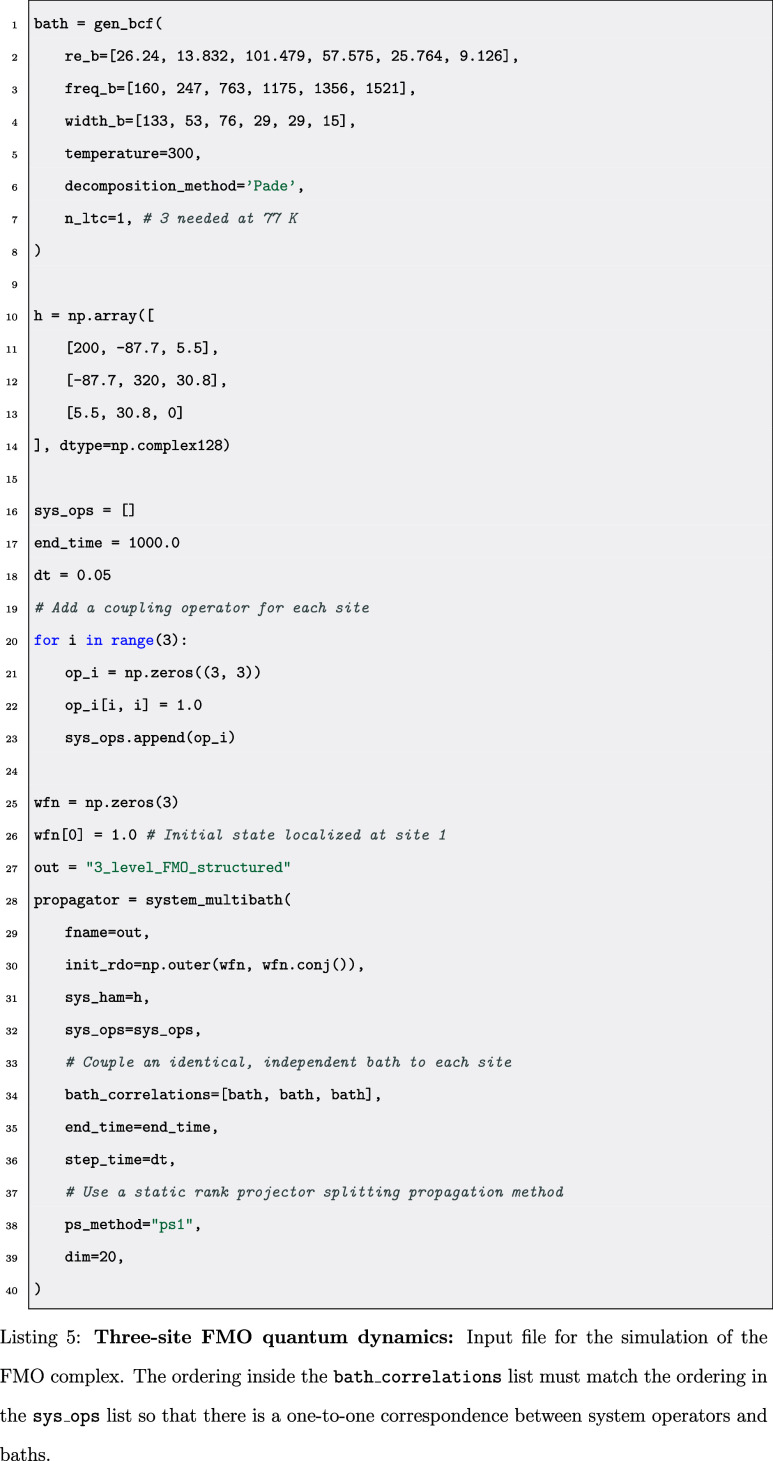


**Figure 6:**
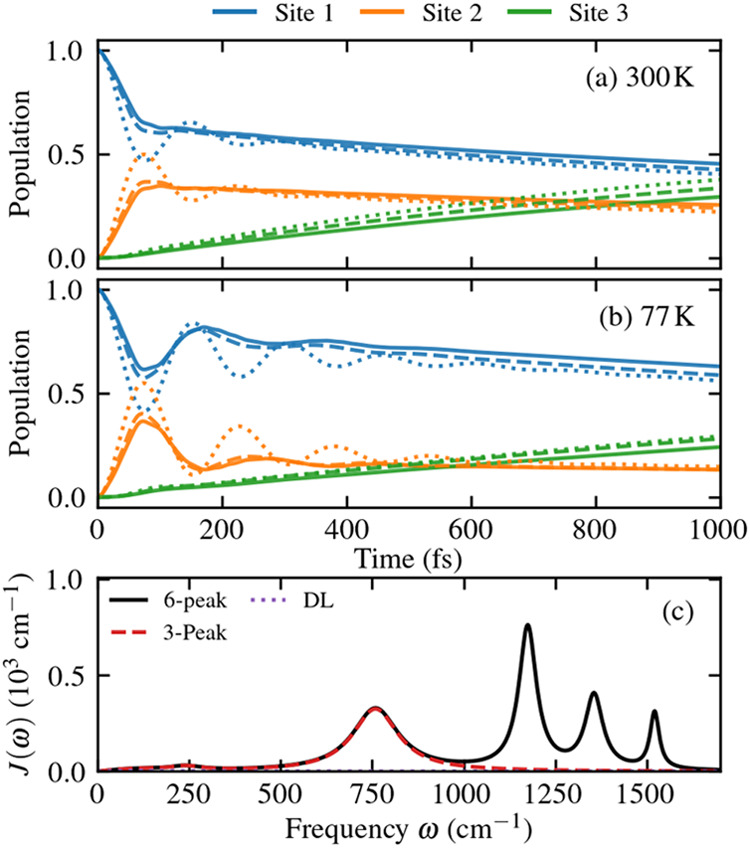
Population dynamics in the FMO complex. Population evolution at (a) *T* = 300 K and (b) *T* = 77 K for three sites, computed with the BTT tensor network decomposition for three different structured spectral densities (solid, dashed, and dotted lines). (c) Three structured spectral densities employed in the simulations.

In Figure 6, we show the dynamics of the FMO complex under the six different baths. Population evolution with the simple Drude–Lorentz bath corresponds to dotted lines; solid lines correspond to the structured spectral density with all six Brownian peaks, and the dashed lines denote the spectral density that uses only the three lowest-frequency Brownian peaks. Significantly different population dynamics are observed for the three different spectral densities, with these differences being far more pronounced at lower temperatures. Higher temperature suppresses population oscillations associated with coherent effects, an expected outcome.^119^ The Drude–Lorentz spectral density produces very divergent dynamics in comparison to the more structured cases, which, notably, have a higher total reorganization energy. For the structured spectral density selected, apparent coherent oscillations are much less pronounced than for the Drude–Lorentz spectral density.

The employment of more realistic, structured spectral densities can have a large impact on the dynamics of the FMO complexes. TENSO’s ability to efficiently simulate systems with these features is a significant asset for treating photosynthetic complexes and related biological systems. The six Brownian peak spectral densities at 77 K yield *K* = 15 features (12 from the BO features and 3 from the low-temperature corrections) per site or 45 total features, which is significantly beyond the applicability of standard HEOM. Note that a thorough assessment of the appropriateness of this and other suggested structured spectral densities for the FMO complex is beyond the scope of this tutorial, and this demonstration seeks only to emphasize their importance and TENSO’s ability to address them.

### 3.5 Example 3: Entanglement Sudden Death

Entanglement between quantum subsystems plays a foundational role in quantum information science and other fields of modern physics.^120–122^ Long-lived, controllable entanglement between subsystems is a key resource in quantum communication, quantum sensing, and quantum computing.^122–125^ Entanglement has also become an important concept in quantum chemistry, offering new insights into electron correlations, molecular dynamics and open quantum systems.^126–128^

However, as a quantum system interacts with its environment, decoherence occurs and entanglement between subsystems decays, presenting a major challenge for hardware design in quantum technologies.^94,95,129^ Density matrix coherence terms often decay exponentially depending on the interaction of an open quantum system with its environment but will remain finite at all times. Entanglement between subsystems, however, may decay to zero within finite time, a subject of intense research known as entanglement sudden death (ESD).^59,130–135^

The simplest system that can display meaningful entanglement is a pair of qubits, equivalently two spin-boson models. As the impact of bath models and non-Markovian effects is of keen interest in this simple model,^136–140^ we use it as an example of the power of TENSO to address problems in quantum information sciences.

We consider two qubits in an initially maximally entangled state, $\left|\psi_{0}\right\rangle =\frac{1}{\sqrt{2}}\left(\left|01\right\rangle -\left|10\right\rangle \right)$ , each interacting with a local Bosonic bath. The system Hamiltonian is given by

27 $$H_{S}=\frac{\hbar \omega_{1}}{2}\sigma_{z}^{\left(1\right)}+\frac{\hbar \omega_{2}}{2}\sigma_{z}^{\left(2\right)}$$

where σ_*z*_ is the usual Pauli operator with the superscript indicating the qubit on which it operates and ω_*i*_ are the frequencies of the qubits. The interaction between the system and baths is given by

28 $$H_{\mathrm{SB}}=\hbar \sigma_{x}^{\left(1\right)}⊗X_{B}^{\left(1\right)}+\hbar \sigma_{x}^{\left(2\right)}⊗X_{B}^{\left(2\right)}$$

where the collective coordinates of each bath are distinguished by superscripts.

We measure entanglement using concurrence. Concurrence is defined for a two-qubit density matrix ρ using the spin-flipped state

29 $$\mathrm{\rho ̃}=\left(\sigma_{y}⊗\sigma_{y}\right)\rho^{*}\left(\sigma_{y}⊗\sigma_{y}\right)$$

where ρ* is the complex conjugate of ρ. We compute the eigenvalues λ_1_, λ_2_, λ_3_, and λ_4_ of the matrix^141^

30 $$R=\sqrt{\sqrt{\rho }\mathrm{\rho ̃}\sqrt{\rho }}$$

and arrange them in decreasing order, λ_1_ ≥ λ_2_ ≥ λ_3_ ≥ λ_4_ then find the concurrence *C*(ρ) as

31 $$C\left(\rho \right)=\max\left(0,\lambda_{1}-\lambda_{2}-\lambda_{3}-\lambda_{4}\right)$$

If *C*(ρ) > 0, the state is entangled, and if *C*(ρ) = 0, the state is separable.

To simulate the pair of qubits, the Hamiltonian and bath parameters for the spin-boson problem demonstrated in Listing 1 are modified as shown in Listing 6. The initial state will be maximally entangled. We will make additional modifications to the bath specifications of the Drude–Lorentz bath, which, unless otherwise noted, has λ = 50 cm^–1^, ω_c_ = 30 cm^–1^, and *T* = 300 K.
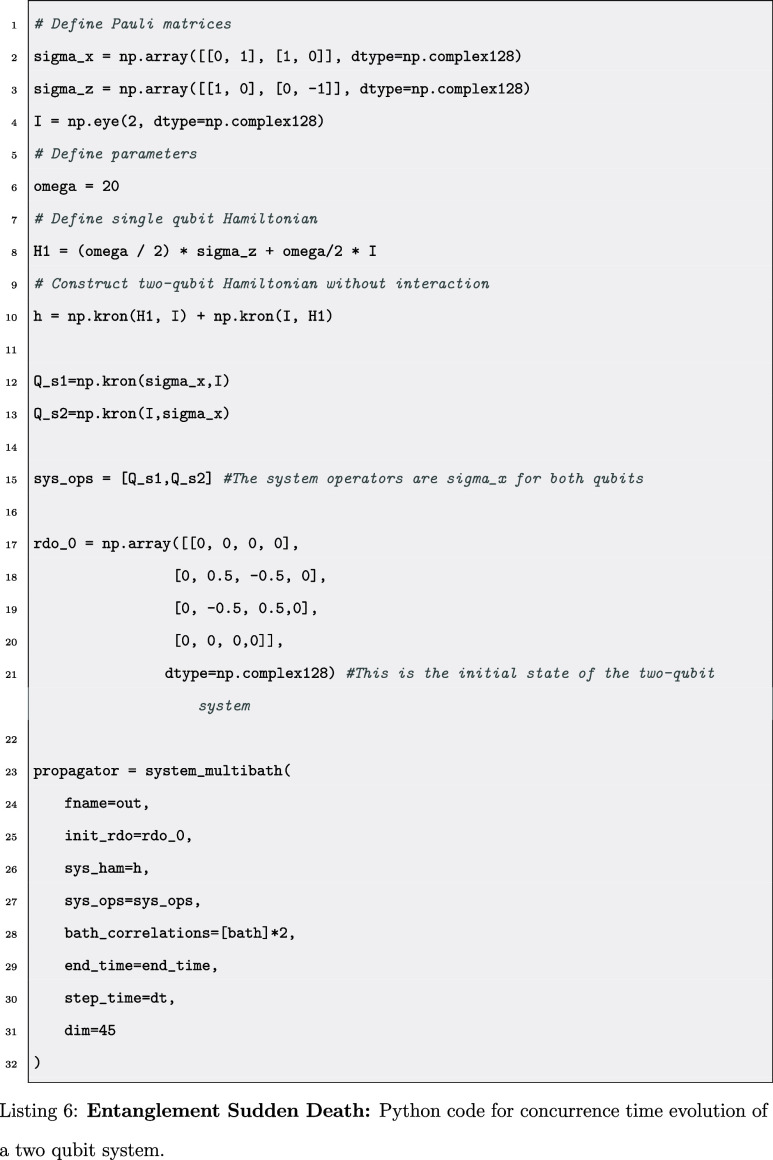


In Figure 7, example population dynamics of the two qubits are displayed at 50 K, where equilibration requires more than 150 fs to complete, and populations are significantly biased in favor of the lower-energy |00⟩ state. In Figure 7b,c, we see that entanglement survival time increases with a lower-temperature bath and smaller reorganization energy. Physically, larger λ indicates stronger system-bath coupling and environmental fluctuations, while a higher temperature *T* amplifies thermal noise, both of which strengthen decoherence channels that rapidly suppress entanglement.

**Figure 7:**
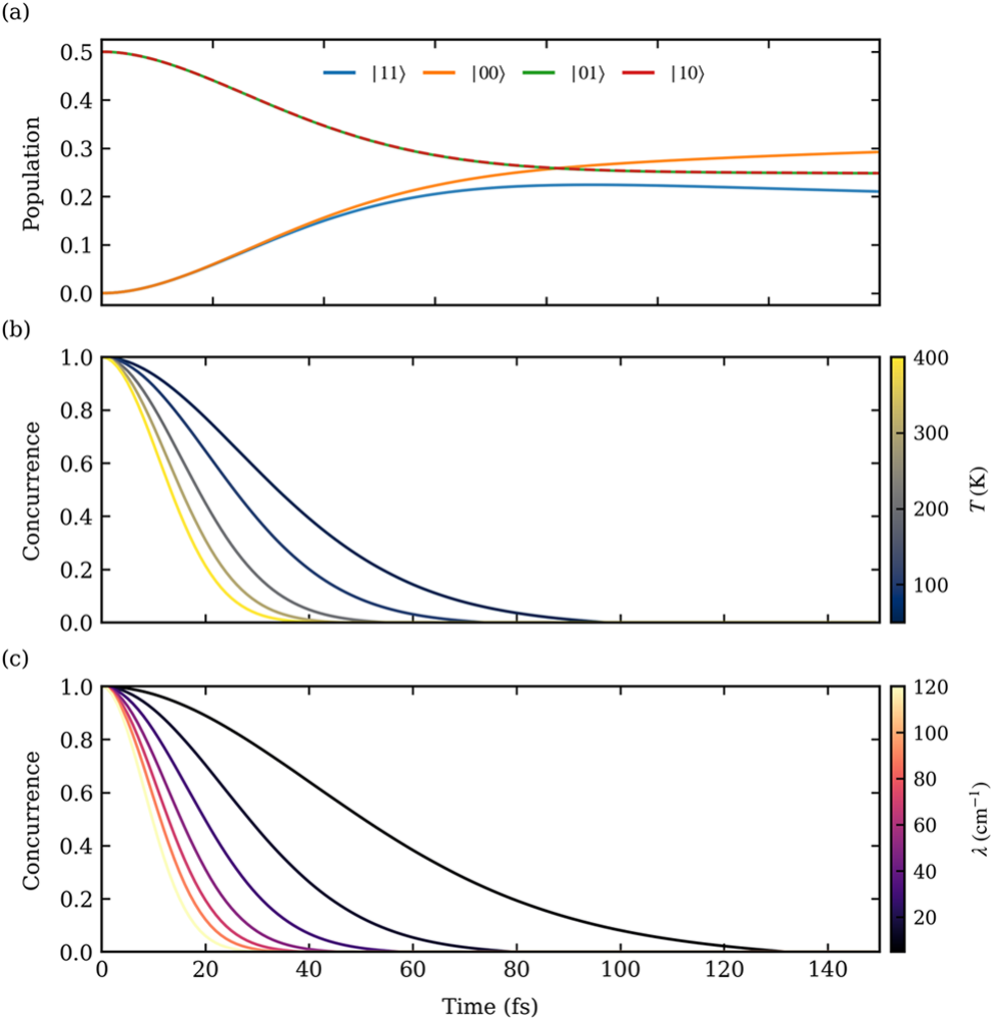
Two-qubit quantum dynamics and entanglement decay. (a) Population dynamics of the two qubits at *T* = 50 K. (b) Decay of the concurrence between the two qubits for varying temperature, with the color scale ranging from low (blue) to high (yellow) values. (c) Decay of the concurrence under variation of the reorganization energy λ, with the color scale ranging from weak (blue) to strong (yellow) system-bath coupling.

For the sake of expediency, the bath was kept simple in this tutorial. The impact of varying even simple bath parameters is generally notable, and TENSO allows for the systematic study of entanglement dynamics under more complicated structured baths for more specialized applications in quantum information sciences.

## 4 Settings Recommendations for Usual Problems

The convergence parameters and propagation methods required depend on the system under study. Here, we provide some general guidance for initial selections. TENSO can either run a calculation with one propagation strategy, requested by passing stepwise_method = “simple” to system_multibath, or run an auxiliary method, specified by auxiliary_ps_method, for an initial period followed by a main method specified by ps_method. We recommend initially addressing a new problem with the default mixed propagation strategy. This runs the adaptive rank projector-splitting ps2 propagation as the auxiliary method to dynamically adjust ranks, followed by fixed rank direct vmf propagation with the default adaptive time step fourth-order Runge–Kutta method for the bulk of the calculation. This is the behavior of TENSO if no information about propagation method is specified.

In some cases, the projector-splitting ps1 propagation method may be preferable to the vmf as the main method. In the case that an adaptive rank is not desired, the default mixed propagation strategy with auxiliary method ps1 and main method vmf is recommended. This helps to avoid regularization errors that often occur with vmf at early times. Possible auxiliary and main method combinations are reviewed in Table 3. Table 4 reviews general recommendations for initial convergence parameter selections, including guidance on how to tighten the parameter to confirm convergence of the calculation. Note that the fixed time step ps1 and ps2 methods require more careful convergence checking with respect to the size of the time step. Parameters not previously discussed include ode_rtol, ode_atol, ps2_atol, ps2_ratio, and max_auxiliary_rank. The first two control the relative and absolute tolerances of the ordinary differential equation integration. The last three control how many singular values are trimmed during the ps2 SVD steps and the maximum rank that ps2 will allow in the tensor network. The default parameters for these five should be well suited to most applications but can be adjusted as needed.

**Table 3:** Comparison of Available Propagation Strategies in TENSO^a^

auxiliary	main	assessment	recommendation
ps2	vmf	adaptive rank, adaptive time step allowed, avoids many vmf regularization errors	recommended method (default)
ps2	ps1	adaptive rank, time step fixed	recommended if adaptive time step is not required or vmf encounters stability problems
ps1	vmf	fixed rank, adaptive time step allowed, avoids many vmf regularization errors	recommended if a fixed rank and adaptive time step is desired
none	ps2	adaptive rank throughout calculation, usually high numerical cost	not recommended
none	ps1	fixed rank, fixed time step	recommended if fixed rank is desired and adaptive time step is not needed
none	vmf	fixed rank, adaptive time step, potential regularization errors	not recommended

a Mixed propagation starts with an auxiliary method, then transitions to a main method. Simple propagation has no auxiliary method. Combinations not shown in this table are never recommended.

**Table 4:** Recommendations for Initial Choice of Important Convergence Settings as well as the Suggested Modification to Make to the Parameter during a Convergence Check

parameter	recommendation	to test convergence
dt	1/20 of the fastest oscillation period	halve
rank	32 (fixed rank methods only)	increase by 2 to 5 as feasible
dim	25	increase by 2 to 5 as feasible
n_ltc	1 for high temperature, 3 for low	increase by 1
ode_rtol	1 × 10^–5^ (default)	decrease by 1 order of magnitude
ode_atol	1 × 10^–7^ (default)	decrease by 1 order of magnitude
max_auxiliary_rank	32 (default)	increase by 5 to 10 as feasible
ps2_atol	1 × 10^–7^ (default)	halve
ps2_ratio	2 (default)	adjust ps2_atol instead

Ideally, convergence can be confirmed with a single calculation in which all of the parameters are relaxed, meaning hierarchy depth, low-temperature corrections, and rank are slightly decreased while the time step size is increased. If no significant changes are observed in the dynamics, then convergence is likely achieved. In the case that this fast test does not indicate convergence, a slight tightening of all parameters in a single additional calculation is advisable. If this is not feasible or does not confirm convergence, it is necessary to increase individual parameters in several different calculations to confirm convergence. When performing convergence testing with respect to individual parameters, we recommend halving time steps, increasing low-temperature corrections by one, and increasing the rank or depth by two to five. The default parameters that control the behavior of the adaptive rank propagator in TENSO should be well suited for most cases but can be adjusted as needed. Table 4 summarizes information about defaults and convergence checking for individual parameters.

## 5 Resources and Scaling

The resource requirements of TENSO depend on the system, the convergence parameters, the propagation strategy, and the hardware. We recommend that TENSO be run on CPUs. Whether available memory or computational time is the limiting factor for TENSO simulations will depend on the particular system, as the computation time required depends not only on the size of the tensor network but also on the relative ease of integrating the equation of motion. Stiff equations of motion require very small time steps, leading to slow calculations.

Parallelization in TENSO is handled by the PyTorch backend, which implements parallel operations for some linear algebra tasks. By default, the backend in TENSO will begin parallel computations on as many cores as are available. We observe that this enhancement is typically capped at 2–4 threads. Some calculations with ps1 or ps2 propagation and very large ranks, on the order of 100, may benefit from additional threads. The number of threads can be controlled by adding _opt.set_num_threads­(n), where *n* is the desired number, in libs/backend.py.

We now use the case of the spin-boson problem considered in Section 3.3 to investigate the scaling of computational time. Many factors influence the computational requirements of TENSO, and it is challenging to formulate simple rules, but some observations can be drawn. Figure 8 compares the wall time for computations with varying bond ranks, hierarchy depths, system sizes, and number of low-temperature corrections for vmf and ps1 computations with identical time steps, either dt = 0.1 or dt = 0.25, propagated for 100 fs with a single thread on an Intel Xeon Gold 6448Y processor. All calculations are performed with the “simple” propagation scheme with fixed ranks. Overall, straight vmf is usually slower than ps1 as it requires extensive regularization at the early stages of the dynamics. Further, the size of the initial time step can have a significant impact on the efficiency of adaptive time step algorithms, in some cases changing whether vmf or ps1 is more efficient.

**Figure 8:**
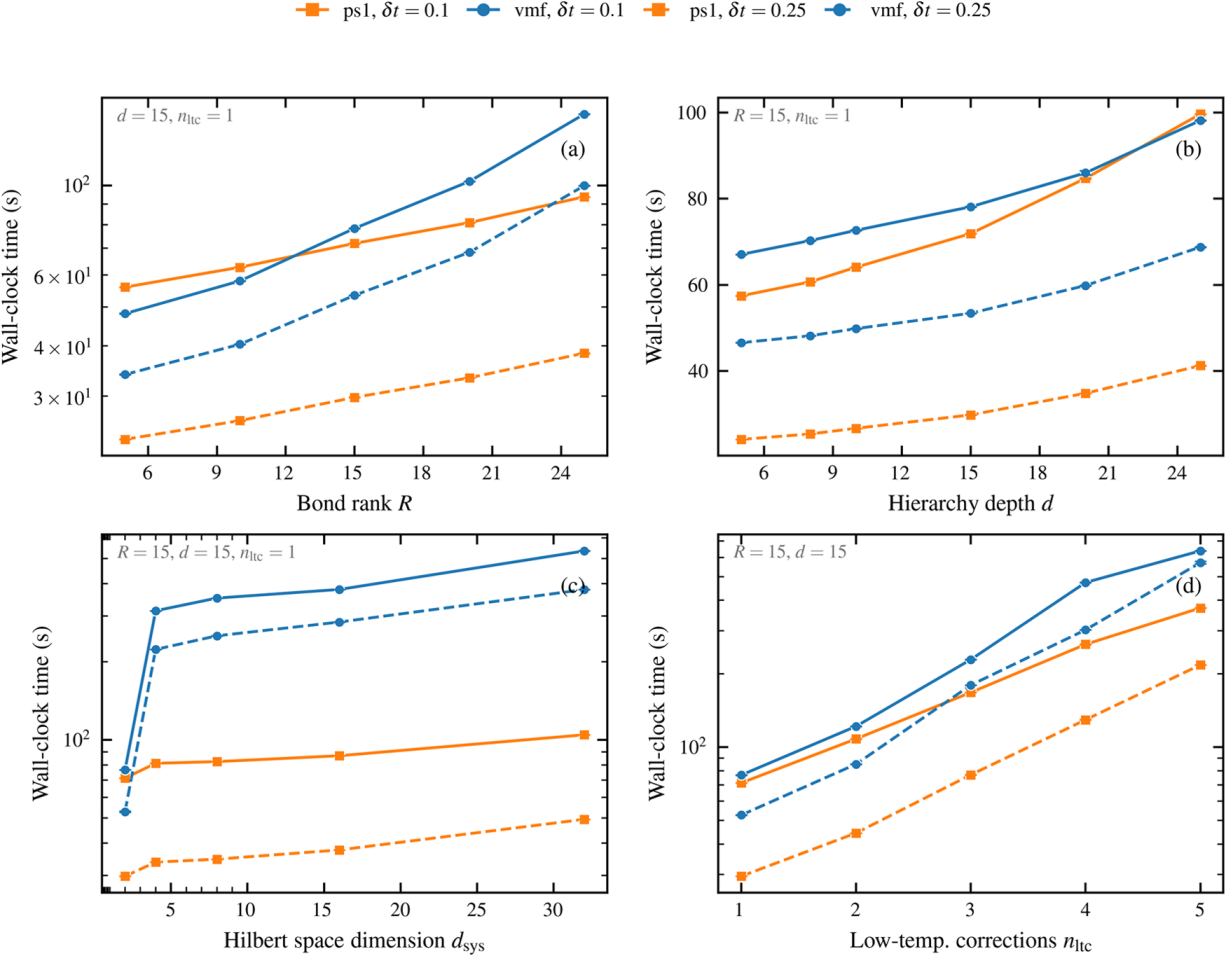
Wall-clock time benchmarks comparing “simple” propagation using the ps1 and vmf integrators at two time steps (δ*t* = 0.1 fs and δ*t* = 0.25 fs) as a function of four key simulation parameters. (a) Scaling with bond rank *R* at fixed hierarchy depth *d* = 15 and *n*_ltc_ = 1. (b) Scaling with hierarchy depth *d* at fixed *R* = 15 and *n*_ltc_ = 1. (c) Scaling with system Hilbert space dimension *d*_sys_ at fixed *R* = 15, *d* = 15, and *n*_ltc_ = 1. Dimension is increased by adding an additional, identical spin. All spins are coupled via σ_*z*_^(*i*)^/2 to a common bath but not to each other. (d) Scaling with the number of low-temperature corrections *n*_ltc_ at fixed *R* = 15 and *d* = 15. The vmf integrator generally exhibits steeper scaling with increasing parameter values compared to ps1, while the larger time step *δt* = 0.25 fs consistently reduces runtime for both methods. The ps1 integrator demonstrates substantially more favorable scaling with *d*_sys_ (c), remaining nearly flat up to *d*_sys_ ≈ 16, whereas vmf shows a sharp increase beyond *d*_sys_ = 2.

Increasing the number of low-temperature corrections and adding additional bath features increases the wall time most dramatically. Increasing the system Hilbert space size by including additional, identical spin degrees of freedom in the system may also result in significant increases in the wall time. In ps1 and vmf, the computational cost increases with Hilbert space dimension *d*, as expected. For vmf, there is an additional computational cost increase with the first additional spin due to the introduction of a new time scale to the adaptive time step method. By contrast, increasing the rank and hierarchy depth leads to significantly more modest increases. The system-bath coupling strength, characterized by the reorganization energy of the bath, also influences the wall time (not shown). Wall time usually increases with coupling strength, but we have observed that the relationship can be nonmonotonic, implying that certain weak-coupling cases are more difficult to integrate than certain strong-coupling cases.

## 6 TENSO Structure

Having demonstrated applications of TENSO in a variety of prototypical quantum dynamics problems, we now discuss those details of the implementation of TENSO that are necessary to understand in order to modify the framework or adjust functionality. Documentation for TENSO is available at https://ifgroup.github.io/pytenso.

The implementation of TENSO contains four layers, as shown in Figure 9. Layer 4 contains interfaces and templates and is the only layer with which most users will need to interact. It is designed to shield users from the more complex layers 1–3. Layer 3 includes implementations of HEOM and MCTDH and routines to set up the tensor network and the propagator that evolve the network in time. Layer 3 builds on the generic functions of layer 2, which provide a general framework for the evolution of any master equation with a sum-of-product form operator. Layer 2 requires libraries such as pytorch and depends on the first layer to provide interfaces to these libraries. Some additional helper functions support multiple layers. We will discuss these layers in ascending order. Note that much of TENSO takes an object-oriented approach, and some familiarity with object-oriented programming is assumed in this section.

**Figure 9:**
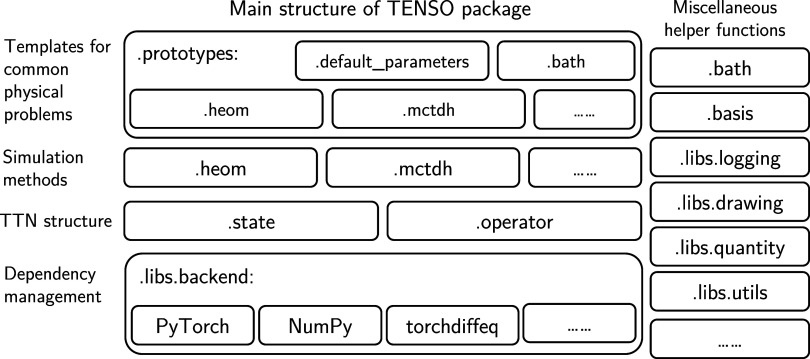
Structure of TENSO. The code TENSO contains four main layers: (1) In tenso.libs.backend, the needed data structures for handling a tensor array are imported from PyTorch and NumPy. (2) The tree tensor network (TTN) structure layer defines the data structures needed for a TTN state and the sum-of-product (SoP) operator, as well as the decomposed master equation and propagation schemes based on the TTN state and SoP dynamics generator. (3) In the simulation method layer, the physical master equations are specified in the TTN decomposition and the SoP dynamics generator. (4) The templates in the prototypes subpackage are interfaces to easily use specific master equation methods. TENSO’s structure allows users to access high-level functions to quickly perform calculations and allows developers easy access to low-level internal structures to build extensions and modifications.

### 6.1 Layer 1: Interfaces with Backend Libraries

In tenso.libs.backend, dependencies and global package settings for PyTorch, NumPy, and torchdiffeq are handled. This includes specifying processor options for PyTorch and importing or implementing integration methods. This file provides significant details on available integration methods other than the default adaptive time step Runge–Kutta.^142^ All TENSO layers above rely on the basic linear algebra routines and pytorch settings in tenso.libs.backend, and a change to settings here is effective everywhere.

### 6.2 Layer 2: TTN Decomposition

The general master equations that TENSO is designed to address are given by eqs 15 and 16. The implementation of the efficient propagation for general master equations is based on two components: the TTN representation of the Ω­(*t*) tensor and the SoP form of the generator of the dynamics, L(t) . The second layer of TENSO implements the tensor network decomposition framework for a generic master equation with a generator in sum-of-products form. Layer 3 above depends on the Node, End, Point, and Frame classes in layer 2 to describe the TTN structure and the associated classes for Model and Propagator.

In the tenso.state module, we implement the data structure for Ω­(*t*). Specifically, tenso.state.pureframe defines the infrastructure needed to represent the underlying graph structure of the tensor network. The Python class Node implements a node in the graph and each instantiated Node object is associated with a core tensor of the decomposition. The class End implements the open bonds in the tensor network. Both Node and End objects keep track of their neighbors through links, edges in the graph, and both are derived from the abstract Point class. A Node object may have more than one link, but an End object must have only one. The class Frame encodes the full graph of the topology of the tensor network. In tenso.state.puremodel, we implement the tree tensor network vector Ω­(*t*) in the class Model. A Model object includes a Frame object describing the TTN structure and data structures to associate a tensor valuation with every Node object in the Frame.

To specify the SoP generator of L(t) , we implement the class SparseSPO in tenso.operator.sparse. This class contains a list of dictionaries, and each dictionary represents an operator product in the sum. The keys are the names of degrees of freedom to operate on, and the values are the corresponding local operators represented by matrices. We also implement the TDVP-based propagator class SparsePropagator in tenso.operator.sparse. This class generates the needed iterators for propagation with a given TTN vector of type tenso.state.puremodel.Model and a given SoP generator of type tenso.operator.sparse.SparseSPO. Specific algorithms such as direct integration and projector-splitting algorithms^14^ are implemented as methods in SparsePropagator.

### 6.3 Layer 3: Implementing Physical Master Equations as a TTN Master Equation

Layer 3 of TENSO builds specific master equations on top of the generic classes provided by the second layer, providing rules to construct specific instances of the tenso.operator.sparse.SparsePropagator and tenso.state.puremodel.Model classes that are relevant to the desired master equations. TENSO currently implements HEOM in tenso.heom and ML-MCTDH in tenso.mctdh. In tenso.heom.eom, we implement HEOM for a system coupled to one bath, and in tenso.heom.meom, we implement HEOM for a system coupled to multiple baths. Regardless of the master equation method, the class Hierarchy in the meom or eom module generates the needed data structure to construct a tenso.operator.sparse.SparsePropagator, as well as a tenso.state.puremodel.Model. The class FrameFactory implements construction of the TTN topologies for Ω­(*t*), including the balanced tensor tree and tensor train topologies.

### 6.4 Layer 4: Templates for Common Problems

Layer 4 of TENSO provides a user-friendly interface, allowing users to avoid interacting with the more complex third layer while initializing a calculation. Interface templates for easy access to HEOM and MCTDH methods relevant to common physical problems are implemented in the subpackage tenso.prototypes. These functions hide all details of the calculation setup to facilitate ease of use. The system_multibath function employed in the examples is located in tenso.prototypes.heom. This interface sets up the system state and propagator, including the tensor network topology that is requested. Similarly, the helper function gen_bcf employed to generate the composite spectral density is implemented in tenso.prototypes.bath. The default values of parameters are also found in this layer in tenso.prototypes.default_parameters.

### 6.5 Miscellaneous and Helper Functions

Additional capabilities are provided by the helper functions. These include algorithms for traversing over a tree to visit all of the nodes and related tasks in tenso.libs.utils and options to use a discrete variable representation (DVR) basis, rather than the default number basis, in the master equations, in tenso.basis. The most important of these auxiliary functions are those that handle the correlation function calculations necessary for computation. The correlation function object Correlation is found in tenso.bath.correlation. The necessary decomposition of the Bose–Einstein distribution is found in tenso.bath.distribution, and tenso.bath.sd handles the definitions of different spectral densities.

## 7 Advanced Features

TENSO is an adaptable code. The preceding sections demonstrated its primary capabilities, features that are critical and available without modification of any part of TENSO. This section outlines how to access niche features and make simple modifications to the code to address specialized applications. We will demonstrate how to implement a custom topology for a TTN, how to modify the metric to adjust the variant of HEOM, and detail some more advanced options for the functions shown in the examples.

### 7.1 Checkpoint Files

It is possible to save a checkpoint file for a calculation. This checkpoint file will include all the information stored in the TTN. This behavior is requested by setting the parameter save_checkpoint_to_file = True when calling system_multibath. Similarly, to load a checkpoint file, set load_checkpoint_from_file = True. The checkpoint file’s name will be the same as the output file, but the extension will be “.pt”. Note that a calculation instructed to read from and write to a checkpoint file will first read and then overwrite the checkpoint.

When loading from a checkpoint file, the initial state, Hamiltonian, operators, and bath correlation passed to system_multibath will not be used for initialization, but they must be present and match those for the loaded checkpoint file. It is not advisable to change any parameters in the propagator except for the time step or end time, noting that propagation is always treated as beginning from time zero. The hierarchy depth and rank settings, in particular, must not be modified.

### 7.2 Custom TTN Implementation

TENSO enables the propagation of TTN of arbitrary order and structure. It offers two predefined TTN structures, the TT and *n*-ary BTT requested by setting frame_method = “train” and frame_method = “treen”, respectively (i.e., frame_method = “tree2”). TENSO supports arbitrary TTN structures specified by defining a root, a list of *K* nodes, and the links between them. A new TTN structure is defined in the FrameFactory class of the file defining the relevant equation of motion, meaning either MCTDH or HEOM (src/tenso/heom/meom.py or src/tenso/mctdh/eom.py for HEOM and MCTDH, respectively) using the methods _new_node­() and add_link­().

Listing 7 illustrates the implementation of a custom TT for a scenario with four total bath features in which the root node is placed in the center of the train rather than at either end. Once the frame “custom” is defined, the corresponding option must be registered in the frame selection logic for system_multibath in src/tenso/prototypes/heom.py (or src/tenso/prototypes/mctdh.py for an MCTDH implementation) as shown in Listing 8. The user can request the custom tree by passing frame_method = “custom”, to system_multibath. The graphical representation of TENSO’s default TT and the “custom” TT is shown in Figure 10, where *n*_*i*_ for 0 ≤ *i* ≤ 3 are bath degrees of freedom, and *i* and *j* are the system degrees of freedom.
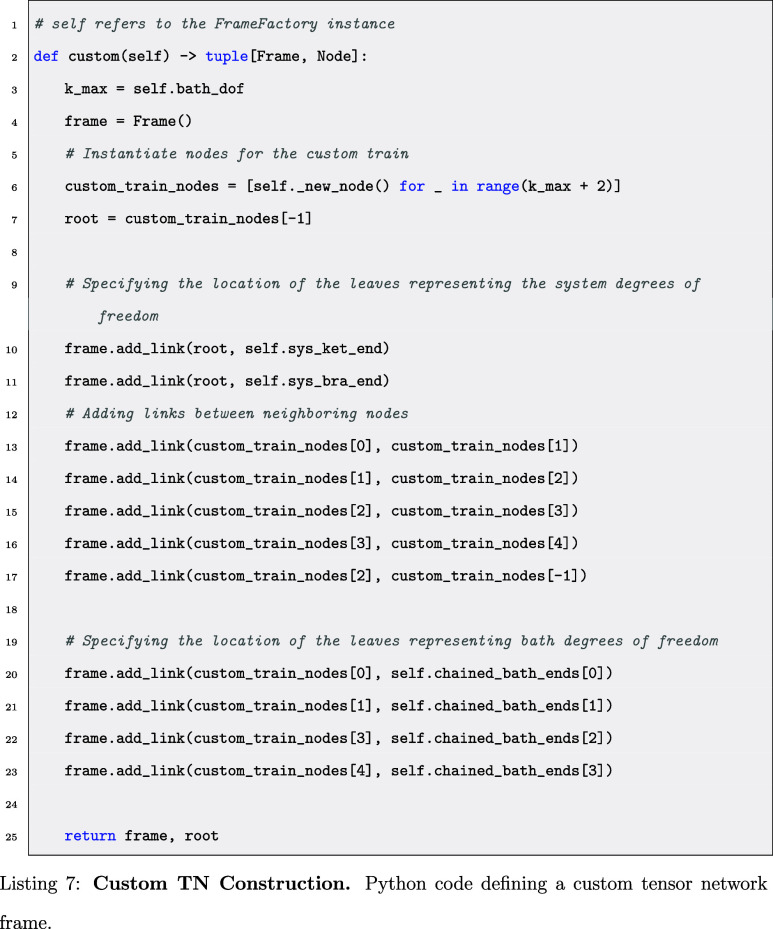
 
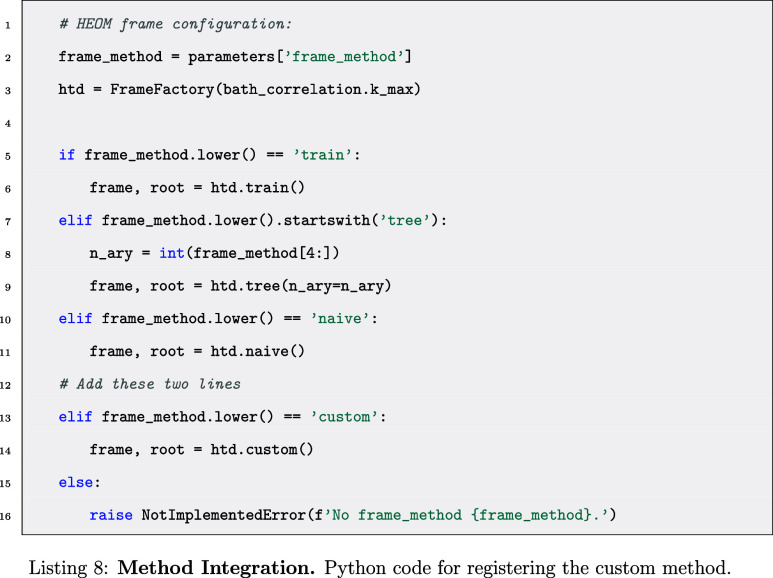


**Figure 10:**
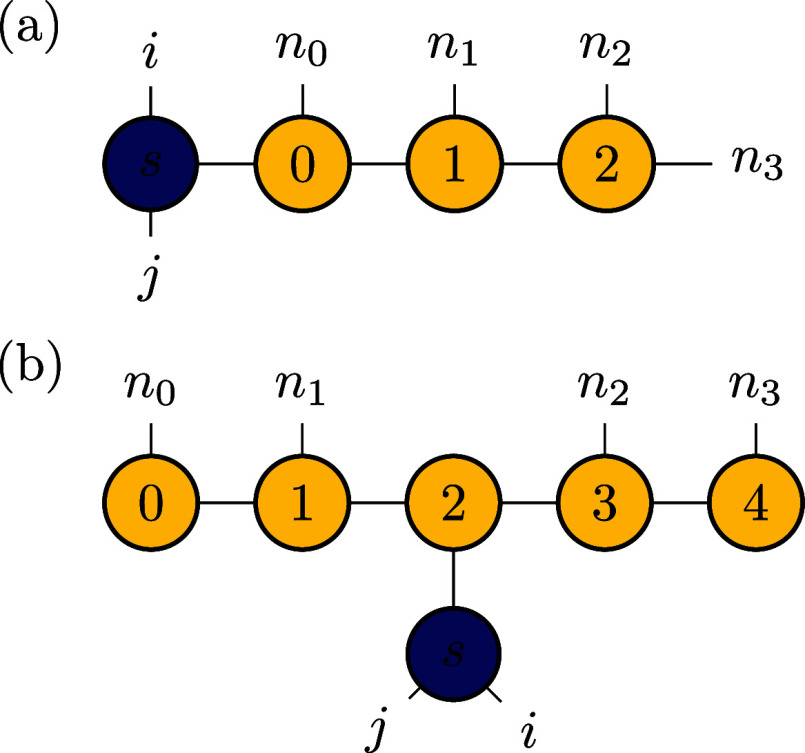
TT structures: (a) Typical TT structure for a 4-feature bath. (b) Custom TT structure for a 4-feature bath. Nodes are represented by circles, system degrees of freedom are *i* and *j*, and bath degrees of freedom are *n*_*i*_ for 0 ≤ *i* ≤ 3.

### 7.3 Custom BCF Implementation

The interface function gen_bcf handles the details of decomposing a BCF into the needed sum of exponentials form when its spectral density consists of DL and Brownian terms. It is possible to add custom spectral densities to src/tenso/bath/sd.py. In TENSO, a spectral density is a class that must extend the abstract SpectralDensity class. A custom spectral density class must implement a constructor, a function returning the spectral density at a given frequency ω, and a function returning a tuple containing lists of the residues and poles. Arguments accepting the new form of spectral density must be added to src/tenso/prototypes/bath.py in gen_bcf along with an option to initialize objects of the custom spectral density type and add them to the list of spectral densities used to generate the BCF.

A custom correlation function can be specified more easily if the complex coefficients *c*_*k*_ and γ_*k*_ are known. The helper function manual_corr_setup in the Correlation class in tenso.bath.correlation performs this function. The process necessary to perform the manual initialization is shown in Listing 9. The bath_simulation produced can be passed to system_multibath as usual. Note that in TENSO *c*_*k*_ have units of energy squared and the γ_*k*_ have units of energy. For real γ_*k*_, only one feature will be introduced. For complex γ_*k*_, the function will introduce a second feature with an exponent of γ_k_^*^. This may lead to redundant features when both γ_*k*_ and γ_k_^*^ are needed to define *C*(*t*).
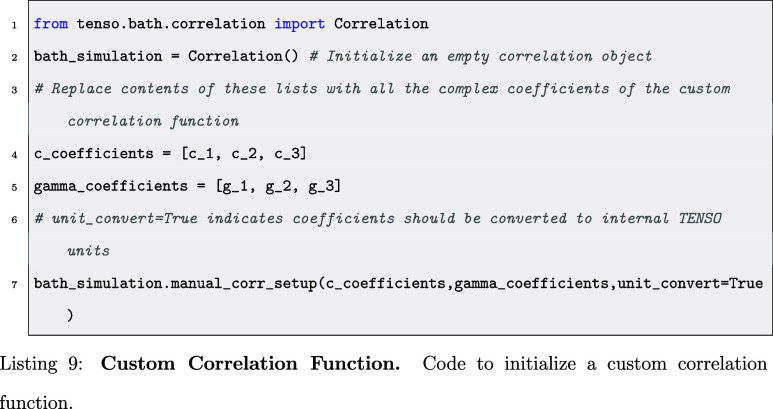


### 7.4 Modifying the HEOM Variant

The metric for the operator of the *k*th bexciton or bath feature is *ẑ*_*k*_|*n*_*k*_⟩= *z*_*k*,*n*_*k*__|*n*_*k*_⟩, where |*n*_*k*_⟩ is the excitation of the *k*th bexciton, *n̂*_*k*_|*n*_*k*_⟩= *n*_*k*_|*n*_*k*_⟩. The commutator [*ẑ*_*k*_,*n̂*_*k*_] = 0, as these operators share a common eigenbasis. The dissipator $D_{k}$ in eq 13 associated with the *k*th bexciton exhibits a clear dependence on the metric. While every metric choice formally leads to identical physical results, different metrics correspond to distinct HEOM variants, and specific choices may generate numerical instabilities. In TENSO, alternative HEOM variants may be realized by implementing a custom metric in the file defining the relevant equation of motion. A new metric for single-bath HEOM is defined in src/tenso/heom/eom.py, as shown in Listing 10. The custom metric is selected with the argument metric = “custom” in the propagator.
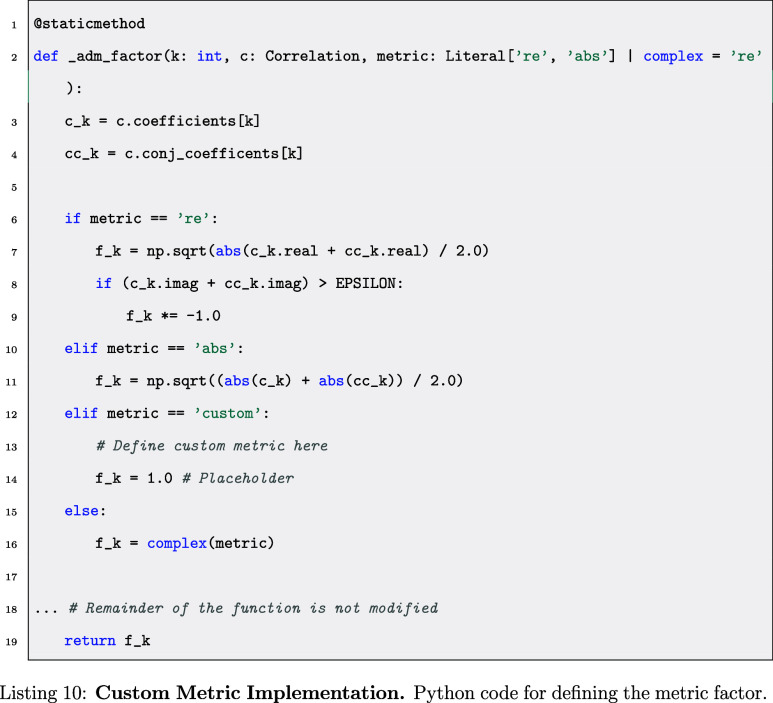


### 7.5 Propagation Scheme and Troubleshooting

We now review several important parameters governing the accuracy and efficiency of TENSO propagation, building on Section 4.

The primary parameter for adjustment of TENSO’s propagation strategy is stepwise_method in system_multibath, which determines the scheme for numerical propagation. By default, the stepwise_method = “mix” method is selected. This approach represents a hybrid scheme, typically combining a projector-splitting algorithm for the initial times, followed by the direct integration method.^14^ The other option is stepwise_method = “simple”, in which case only one propagation method is used for the entire simulation, selected by setting the argument ps_method equal to “vmf” for variable-mean field direct propagation, “ps1” for the static rank projector-splitting method, and “ps2” for the adaptive rank projector-splitting method.

When “mix” is selected, the initial phase of the propagation is controlled by the system_multibath parameter auxiliary_ps_method. This specifies the propagation of the algorithm. The default is the adaptive rank ps2, and the max_auxiliary_rank parameter sets the upper bound on the tensor rank. When the rank limit is reached, the propagation method changes to the method specified by the ps_method parameter, with “vmf” being the default.

The accuracy of the nonadaptive rank projector-splitting algorithm, ps1, is determined by the tensor rank. In a mixed propagation beginning with ps2 and transitioning to ps1 or vmf, the second method uses the final ranks selected by ps2. Otherwise, the value specified by the parameter rank is used. For the rank-adaptive ps2 propagator, accuracy and efficiency are determined by the maximum size of the singular values (SVs) truncated during the algorithm. The argument ps2_atol defines the truncation threshold for the singular values. To mitigate the loss of significant correlations during truncation, a fraction of discarded SVs is reincorporated, with this fraction determined by the ps2_ratio. The direct integration vmf method relies on standard ordinary differential equation solvers with the method determined by the ode_method parameter. The default integration method is dopri5, a fifth-order Runge–Kutta method, with additional options detailed in tenso.libs.backend. The error tolerances of the integrator are controlled by absolute, ode_atol, and relative, ode_rtol, tolerances.

The direct integration scheme also requires the regularization of the equations of motion. The relevant parameters control the tolerance, vmf_atol, method, vmf_reg_method, and procedure, vmf_reg_type. Regularization is essential for the time evolution of the nonroot tensors, which require the inversion of a matrix that may be ill-conditioned. The vmf_atol parameter acts as a regularization term added to the diagonal elements of the ill-conditioned matrix to ensure that it is invertible. This regularization technique is always employed when the propagation scheme is set to the vmf. Note that seriously ill-conditioned matrices are most likely to arise at the start of the simulation, which is why the default mixed propagation scheme does not start with the vmf method.

The reliability and accuracy of TENSO’s tensor network propagation strategies depend on interactions between control parameters and on the structure of the tensor network. TTN-HEOM equations of motion and their implementation into TENSO admit arbitrary tensor tree structures with tensors of any order; in practice, however, some choices may outperform others. There is no means to predict which tree structures may be more favorable. TENSO’s default balanced binary tree combined with mixed ps2 to vmf propagation is the recommended initial treatment of a system. In the case that a simulation is unsuccessful, it is possible that the problem is unsuitable for a tensor network compression or it is possible that different control parameters are required for stable propagation. Decreasing the time step, changing between vmf, ps1, or ps2 propagation, or selecting an alternative integrator from src/tenso/libs/backend may improve the situation. Such cases should be carefully scrutinized for the convergence.

### 7.6 Modifying Default Units

TENSO’s default units are defined in prototypes/default_parameters.py. The units that TENSO assumes when reading input from system_multibath and bath_correlation are defined in a dictionary, default_units, where keys are properties and values are strings corresponding to physical constants imported from SciPy, with the available list detailed in libs/quantity.py. Options for energy units include the default inverse centimeters, “/cm”, joules, “J”, electronvolts, “eV”, and millielectronvolts, “meV”. Options for time include the default femtoseconds, “fs”, seconds, “s”, and picoseconds, “ps”. Inverse temperature, not temperature, is defined, with the only option being the default, inverse Kelvin, “/K”.

TENSO converts input into internal units and then scales energy and time by the factor associated with “unital_energy” in the default_units dictionary. Specifically, any units of time or inverse temperature will be multiplied by this factor, and any units of energy will be divided by this factor. The default value is 1000. Adjusting this value may alleviate numerical problems when dealing with energies that are very large or very small in absolute terms.

## 8 Conclusion

By combining the hierarchical equations of motion with tensor network representations, TENSO provides an efficient and systematically improvable framework that alleviates the curse of dimensionality inherent in conventional HEOM simulations. The resulting approach retains the formal exactness of HEOM while extending its applicability to complex spectral densities and arbitrary Hamiltonians, including those with explicit time dependence.

We demonstrated the versatility of TENSO through a series of representative examples ranging from structured spin–boson models and driven two-level systems to the Fenna–Matthews–Olson complex to scenarios exhibiting entanglement sudden death. In each case, TENSO was able to capture rich dynamical behavior with reduced computational overhead, highlighting the efficiency of the TTN-based propagation scheme.

A key strength of TENSO lies in its modular and extensible design, providing both accessibility for nonspecialists and flexibility for advanced users developing new approaches. By making the underlying tensor operations and graph-based decomposition structures available for advanced users, TENSO serves not only as a powerful simulation tool but also as a platform for further method development. It can easily be adapted to employ specialized and efficient correlation function decomposition schemes such as ESPRIT^143–145^ and A4 ^146^ as needed, for instance, to address low-temperature cases difficult for traditional decomposition schemes. TENSO’s cost-saving strategy for HEOM is compatible with alternative approaches to reduce the computational cost of capturing structured baths, including pseudomode,^115,147–149^ two-particle approximation methods,^150,151^ and other strategies. The combination of TENSO with these additional strategies offers fertile ground for the development of increasingly powerful computational strategies for HEOM-based simulations. Future studies will investigate the limits of the tensor network decomposition of HEOM.

Taken together, its features position TENSO as a general and efficient framework for simulating quantum dynamics in complex environments.

## Data Availability

TENSO is available under the MIT license. The python code for TENSO, including examples, is available at https://github.com/ifgroup/pytenso. The data underlying this study are available in the published article.
